# The evolutionary history of sharp- and blunt-snouted lenok (*Brachymystax lenok *(Pallas, 1773)) and its implications for the paleo-hydrological history of Siberia

**DOI:** 10.1186/1471-2148-8-40

**Published:** 2008-02-06

**Authors:** Elsa Froufe, Sergey Alekseyev, Paulo Alexandrino, Steven Weiss

**Affiliations:** 1 Centro de Investigação em Biodiversidade e Recursos Genéticos (CIBIO/UP), Campus Agrário de Vairão, 4485-661 Vairão, Portugal; 2 Faculdade de Ciências, Universidade do Porto, Praça Gomes Teixeira, 4009-002 Porto, Portugal; 3 N. K. Kolzov Institute of Developmental Biology (IDB), Russian Academy of Sciences, 117334, Moscow, Vavilova 26, Russia; 4 Karl-Franzens Universität Graz, Institut für Zoologie, Universitätsplatz 2, A-8010 Graz, Austria

## Abstract

**Background:**

Broad-scale phylogeographic studies of freshwater organisms provide not only an invaluable framework for understanding the evolutionary history of species, but also a genetic imprint of the paleo-hydrological dynamics stemming from climatic change. Few such studies have been carried out in Siberia, a vast region over which the extent of Pleistocene glaciation is still disputed. *Brachymystax lenok *is a salmonid fish distributed throughout Siberia, exhibiting two forms hypothesized to have undergone extensive range expansion, genetic exchange, and multiple speciation. A comprehensive phylogeographic investigation should clarify these hypotheses as well as provide insights on Siberia's paleo-hydrological stability.

**Results:**

Molecular-sequence (mtDNA) based phylogenetic and morphological analysis of *Brachymystax *throughout Siberia support that sharp- and blunt-snouted lenok are independent evolutionary lineages, with the majority of their variation distributed among major river basins. Their evolutionary independence was further supported through the analysis of 11 microsatellite loci in three areas of sympatry, which revealed little to no evidence of introgression. Phylogeographic structure reflects climatic limitations, especially for blunt-snouted lenok above 56° N during one or more glacial maxima. Presumed glacial refugia as well as interbasin exchange were not congruent for the two lineages, perhaps reflecting differing dispersal abilities and response to climatic change. Inferred demographic expansions were dated earlier than the Last Glacial Maximum (LGM). Evidence for repeated trans-basin exchange was especially clear between the Amur and Lena catchments. Divergence of sharp-snouted lenok in the Selenga-Baikal catchment may correspond to the isolation of Lake Baikal in the mid-Pleistocene, while older isolation events are apparent for blunt-snouted lenok in the extreme east and sharp-snouted lenok in the extreme west of their respective distributions.

**Conclusion:**

Sharp- and blunt-snouted lenok have apparently undergone a long, independent, and demographically dynamic evolutionary history in Siberia, supporting their recognition as two good biological species. Considering the timing and extent of expansions and trans-basin dispersal, it is doubtful that these historical dynamics could have been generated without major rearrangements in the paleo-hydrological network, stemming from the formation and melting of large-scale glacial complexes much older than the LGM.

## Background

Our knowledge on the evolutionary history of north temperate fishes has been fundamentally altered due to the advent and application of broad-scale phylogeography [1-4]. Phylogeographic investigations of freshwater fishes in Europe are numerous and inferences drawn on the history of intraspecific lineages often relate to how river courses and their accompanying catchment basins dynamically change through several glacial epochs [e.g., [5,6]]. For cold tolerant fishes such inferences can be complex. Genetic lineages can be distributed mosaically among basins, reflecting repeated population expansions and contractions across the shifting colonization corridors that have resulted from river capture events, the formation and dynamics of pro-glacial lakes and fluctuating levels and salinities of seas [7-9]. Despite relatively sound knowledge of European glaciation and attempts to find common patterns, phylogeographic scenarios are often species specific.

There are few similar studies in Siberia and far less certainty concerning the extent of glaciation and paleohydrological stability [10]. One of the first broad-scale phylogeographic studies in Siberia reported that genetic lineages of grayling (genus *Thymallus*), corresponded to major Siberian river systems (e.g. Amur, Lena, Enisei) [11]. The study also supported that grayling had been extirpated from Lake Baikal during the early to mid-Pleistocene as the result of some climate-induced environmental perturbation. Subsequently, grayling were able to recolonize Lake Baikal when its waters over spilled forming a new outlet into the Enisei basin, 110,000 to 450,000 years ago [11]. The authors speculated that this event might relate to highly controversial hypotheses concerning the paleo-climate in Siberia. Most geologists consider Siberian glaciation to have been rather limited based on the modeling of sparse precipitation during the Pleistocene (minimum model) [12]. However, field evidence supports extensive glaciation along the polar continental shelves and coastal Pacific lowlands (maximum model) [13]. Such ice sheets would have blocked north flowing rivers and created a series of pro-glacial lakes. Evidence for such blockage has been presented for the Ob and Enisei systems [14,15].

Furthermore, interior mountain regions (e.g. Trans-Baikalian) were glaciated perhaps above 1000–1200 m. However, many potential refugia for cold tolerant organisms must have existed in central and east Siberia, north of interior mountain systems, as supported by phylogeographic patterns found in grayling from the Lena basin [16]. Siberian glacial scenarios, however, are much in dispute, especially for the last glacial maximum (LGM) [17]. Recent studies reflect an appreciation for the region's paleohydrological dynamics and its effects on organismal history [6,18-21]. Nonetheless, no study has of yet covered the majority of Siberia where four of the world's ten largest rivers occur (Ob, Lena, Enisei, and Amur).

**Figure 1:**
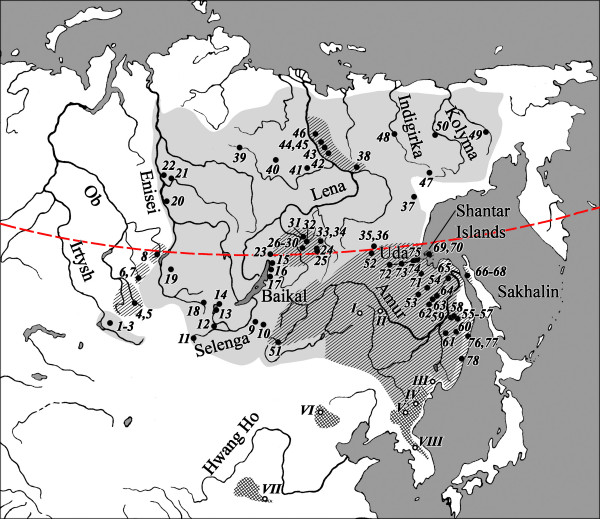
**Ranges and sampling locations of sharp- andblunt-snouted lenoks**. Ranges of sharp- and blunt-snouted lenoks compiled from data and literature. Grey shading, sharp-snouted lenok; left hatch, blunt-snouted lenok; crosshatch, form uncertain. Korean and Chinese parts of the range follow Kim (1972) and Li (1985). Arab numerals and solid circles correspond to sample sites of this study, listed in Table 1, Roman numerals and open circles – to sample sites for the external data from China and Korea: I – Songhua (Sungari), II – Woniuhe, III – Suifenhe (Suifun), IV – Tumen, V – Yalu, VI – Yellow River (Hwang Ho) basin, VII – Tuligenhe (China – Yingzhe *et al*, 2006), VIII – Hanjiang (Korea). The red-dotted line corresponds to 56° N.

The Asian endemic salmonid fish *Brachymystax lenok *occurs in all major Siberian river systems (Figure 1) and thus can serve as a phylogeographic model for assessing paleohydrological events. Lenoks occur in two morphological forms, differing in the length and shape of their snouts as well as a number of external morphological and osteological characters. The so-called sharp- and blunt-snouted lenoks are viewed as either one species complex *B. lenok *represented by two infraspecies [22], or as two nominal species. Morphological variation within each form exists among major basins [22,23] and in zones of sympatry F1 hybrids are found [[24-26], Additional File 1]. Interestingly, longitudinal clines in morphological characters led to the hypothesis of countercurrent dispersal, with sharp-snouted lenok expanding from the west and blunt-snouted from the east [23]. Such dispersal, combined with character displacement in contact zones is thought to have resulted in the formation of clines [22,23].

Analysis of allozyme variation supported limited gene flow between the two forms [26,27]. Several enzyme systems can distinguish sharp- and blunt-snouted lenoks within basins, particularly in sympatry, but no alleles are consistently diagnostic throughout Siberia. Moreover, the range-wide pattern of allelic distribution for di-allelic loci is complex with no consistency as to which alleles are fixed or found in high frequencies within a form among all river basins. These results were interpreted as evidence of extensive hybridization and gene exchange in the past, with the present structure of the genus formed via "multiple hybrid speciation" [22], though such interpretation received criticism [27].

Considering the interesting evolutionary scenario of sharp- and blunt-snouted lenoks as well as their widespread distribution in Siberia, we proposed a combined genetic and morphological approach to test the existing hypotheses of counter-current dispersal, hybridization and gene flow between the two forms. Concomitantly, we aimed to use lenok as a phylogeographic model for further understanding the paleohydrological dynamics of the Pleistocene in Siberia.

## Results

**Figure 2:**
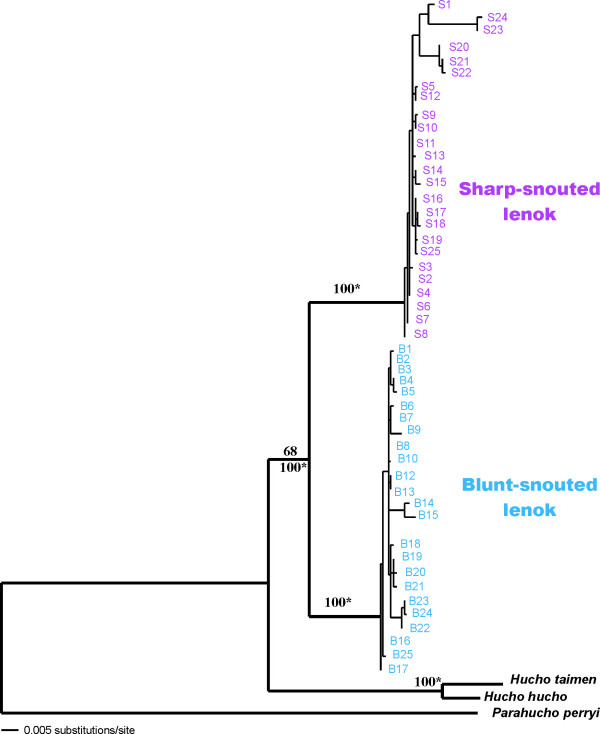
**ML tree based on CR and ND1 sequences**. Tree derived from a ML search using the Tamura-Nei model (TRN+G+I) for the CR and ND1 sequences combined. All analyses (NJ, MP, and ML) gave similar estimates of relationships. For the major clades, bootstrap values (over 50%) are shown for ML (above); MP (with gaps) (below, left) and NJ (below, right). 100*means that all bootstrap values are higher than 95. The tree is rooted with *H. hucho*, *H. taimen *and *P. perryi*.

The final alignment included 494 bp of the mtDNA control region (CR) (*N *= 151), 987 bp of the NADH-1 gene (ND1) (*N *= 142), and 1481 bp (*N *= 114) with both genes combined. There was no significant deviation from base frequency homogeneity across taxa for either gene. In lenok the transition/transversion ratio was 2.56 for the CR, which revealed one 2-bp and four 1-bp indels, with outgroup taxa included. For the coding ND1 there were no indels nor amino acid changes. Neither transitions nor transversions were saturated in either gene segment, including third codon positions. There were 33 variable sites for the CR, 30 of which were parsimony informative (excl. indels) and 208 variable sites (109 parsimony informative) for the ND1. Pairwise sequence divergence within *B. lenok *ranged from 0 to 3.0% (CR) to 0 to 6.2% (ND1). In all analyses two monophyletic groups are identified corresponding to blunt- and sharp-snouted lenoks (Figure 2). Net divergence between groups ranged from 1.7% (CR) to 4.7% (ND1), while within lineage divergences ranged from 0.2% to 0.7%. Net divergences between outgroup taxa and lenok varied with the two genes: 4.3% (CR) or 7.7% (ND1) for *H. hucho*, 5.4%(CR) or 7.4% (ND1) for *H. taimen*, and 7.6% (CR) or 13.3% (ND1) for *P. perryi*.

**Figure 3:**
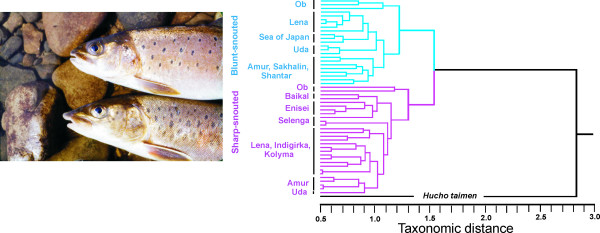
**UPGMA dendrogram of external morphological and osteological characters**. UPGMA dendrogram of lenok populations (N = 50) based on 46 external morphological and osteological characters. H. taimen is added to the analysis. Photograph of both sharp- and blunt-snouted lenoks’heads. Population numbers corresponding to those in Table 1 can be found in Additional File 11 and Additional file 12.

**Figure 4:**
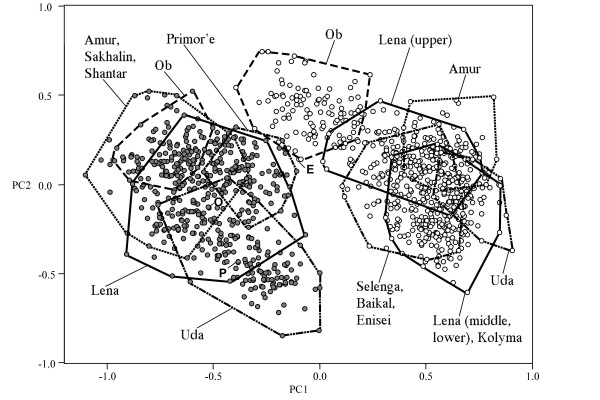
**Scatterplot of PCA factors based on morphological and osteological characters**. PCA scatterplot of sharp-snouted (open circles) and blunt-snouted (filled circles) lenoks from different parts of Siberia and the Far East of Russia based on 46 external morphological and osteological characters.

The phenogram based on external morphological and osteological characters parallels the genetic results revealing two clusters corresponding to sharp- and blunt-snouted lenoks (Figure 3). Similarly, a plot of the first two factors of a Principal Component Analysis (PCA) reveals two clusters clearly representing the two forms (Figure 4). Only the position of sharp-snouted lenok from the Ob basin, intermediate along the first factor (PC1) between blunt-snouted lenok and all other sharp-snouted individuals prevents 100% diagnosis of all individuals to a form. Size effects were assumed to be minimal as there was no correlation between PC1 and size (Additional File 2) and mean total length of individuals analyzed in both forms was nearly identical (sharp-snouted 41.2 cm; blunt-snouted 41.6 cm). Application of a Discriminant Function (DF) produced from a 25% random sample of the data set resulted in 99.9% and 99.6% correct identification for the blunt- and sharp-snouted lenok, respectively. DF values for the misclassified individuals (two Ob basin sharps and 5 Primor'e region blunts) were intermediate to the ranges of values defining each form.

### Haplotype Networks

**Figure 5:**
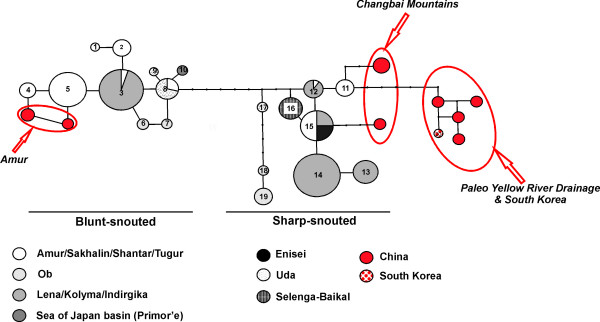
**Parsimony networks of CR haplotypes** Parsimony network (95%) of CR haplotypes observed in blunt- and sharp-snouted B. lenok from Siberia including the nine haplotypes from GenBank (shown in red), representing Chinese and Korean samples. Circle size is proportional to observed haplotype frequencies and black points represent unobserved haplotypes. [The name B. tumensis was suggested for blunt-snouted lenok, but this name was first given to fish bearing the haplotype from the Tumen River, shown in this network to group with the sharp-snouted mtDNA lineage.]

The CR network revealed 19 haplotypes, 10 in blunt- and 9 in sharp-snouted lenok (Figure 5; Additional File 3). Maximum pairwise divergence was considerably less within blunt-snouted (six steps) compared to sharp-snouted (twelve steps) lenok (Figure 5). Both forms revealed at least one haplotype shared between the Lena and Amur drainages supporting dispersal of some kind between these basins. Both forms also revealed private haplotypes at the eastern (Uda) as well as western (Ob) edge of their distributions, supporting isolation of these drainages.

The addition of 15 GenBank sequences (from China and Korea) to the CR network revealed a significant finding. While several haplotypes from mainland China clustered with both our blunt- and sharp-snouted clades, the remaining haplotypes from Korea and the Paleo-Yellow River formed a distinct cluster, a minimum of seven steps divergent from our sharp-snouted clade (Figure 5). Despite the lack of morphological data it is likely that the first two groups of haplotypes represent blunt-snouted and sharp-snouted lenoks, respectively.

**Figure 6:**
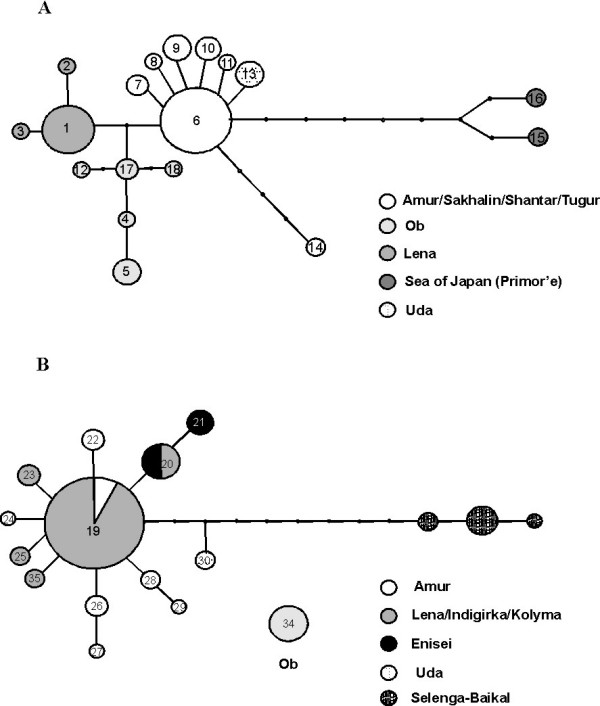
**Parsimony networks of ND1 haplotypes**. Parsimony networks (95%) of ND1 haplotypes in (A) blunt-snouted and (B) sharp-snouted lenok. Circle size is proportional to the observed haplotype frequencies and black points represent unobserved haplotypes.

Parsimony analysis of the ND1 gene revealed nearly twice the number of haplotypes (N = 35) as the CR, suggesting a considerably higher substitution rate for this coding gene seqment. Two separate networks were revealed, in addition to one highly divergent sharp-snouted haplotype from the Ob basin (Figures 6A and 6B; Additional File 4). Both networks revealed at least one star-like cluster of haplotypes, reflecting demographic expansions of populations primarily from the Amur (blunt-snouted) and the Amur and Lena (sharp-snouted) basins. Each network also revealed one or more highly divergent groups, spanning 12 steps in blunt- and 13 steps in the sharp-snouted lenok.

The blunt-snouted lenok ND1 network (18 haplotypes) contained no shared haplotypes between the Lena and Amur catchments in contrast to analysis with the more slowly evolving CR. Some Amur haplotypes were shared with the Tugur River and/or one of the island populations, whereby the Shantar Islands also revealed a unique haplotype reflecting a degree of long-term isolation. Considerably longer periods of isolation were suggested for both the extreme eastern (Ob) and western (Sea of Japan) regions of the blunt-snouted lenok's distribution. The Ob basin revealed five unique haplotypes that were a minimum of two steps from either the Lena's or Amur's most common haplotype. In the Sea of Japan basin, two very distinct haplotypes (ND15 & ND16) were found, both a minimum of eight steps from any other in the network.

The sharp-snouted ND1 network (17 haplotypes) revealed one presumably ancestral haplotype (ND17) shared among populations throughout the Lena, as well as locations in the Indigirka, Kolyma and Amur basins. This haplotype is central to a star-like cluster of 11 haplotypes, each one or two base pairs divergent from ND17. Unique haplotypes were found in the Amur, Tugur, Enisei, and Uda basins. Three haplotypes from the Selenga-Baikal basin (ND31, 32, 33) form a highly divergent group, suggesting a refuge or isolation, not reflected in data sets from other salmonid fishes [11]. The Ob basin is fixed for one, highly divergent haplotype (ND34) beyond the 95% parsimony limit defining the network, which, together with the results of the blunt-snouted lenok, demonstrate long-term isolation of this basin.

### Among basin comparisons (Amova & pairwise differences)

For both lenok forms, the among group variance (Φ_CT_), representing differences between major ocean basins (Arctic and Pacific) was minimal (or negative) and statistically non-significant, while the largest and highly signficant component (Φ_SC_) represented the among river basin variance (Additional File 5). A overview this among basin variability can be provided with a table of pairwise differences, which demonstrates relatively large average differences among most pairs of basins, except those that share some haplotypes (Additional File 6), such as the Amur and Lena (both forms) or Lena and Enisey (sharp only). Within basin variation is mostly much lower, except for those basins harboring multiple divergent haplotypes such as the Primor'ye and Ob basins for blunt-snouted lenok, and the Enisei or Tugur/Uda region for sharp-snouted lenok.

### Mismatch Analysis

The CR pairwise mismatch distribution was uni-modal for blunt-, and bi-modal for sharp-snouted lenok (Additional File 7). Removal of regionally restricted haplotypes (Ob and Uda basins) in sharp-snouted lenok, reflecting population subdivision allowed analysis of uni-modal distributions for both forms, and these distributions both differed significantly from those expected for stable population sizes (K-S tests; P < 0.0001). Additionally, using the least-squares approach, there was no significant difference between observed and simulated data under an expansion model for either form (blunts: SSd, *P *= 0.108; Harpending's raggedness index, *P *= 0.116; sharps: SSd, *P *= 0.1680; Harpending's raggedness index, *P *= 0.1000).

The ND1 mismatch distribution was multi-modal for both forms (Additional File 8). Similar to the CR analysis, removal of geographically distinct haplotype groups allowed analysis of uni-modal distributions, representing the Amur and Uda basins for blunt-snouted, and the Amur and Lena basins for sharp-snouted lenok. Again, observed mismatch distributions were significantly different from that expected under stable population models (K-S tests; *P *< 0.0001), but the data did not fit the expansion model either.

**Figure 7:**
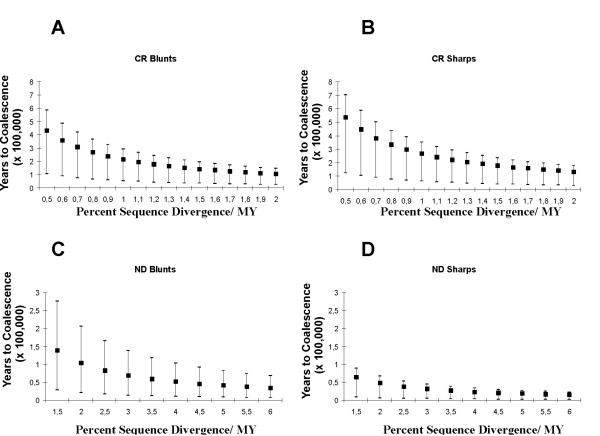
**Coalescence estimates for the CR and ND1 genes**. Coalescence estimates for the CR, based on τ and its 95% CI shown for a range (0.5%–3%) of divergence rates for **(A) **blunt-snouted and **(B) **sharp-snouted lenok. Coalescence estimates for the ND1 gene based on τ and its 95% CI shown for a range (1.5%–6%) of divergence rates for **(C) **blunt-snouted and **(D) **for sharp-snouted lenok.

Using estimates for the expansion parameter *tau*, along with divergence rate estimates ranging from 0.5 to 3% per million years for the CR and 1.5 to 6% for the ND1, we estimated the mean age of expansion for both genes in both forms (Figure 7A–D). Mean estimates of expansion times, regardless of the gene or presumed substitution rate clearly relate to periods in the mid- to late Pleistocene (50,000 to 400,000 year ago) but in all cases earlier than the LGM (18,000 years ago).

### Microsatellites

**Figure 8:**
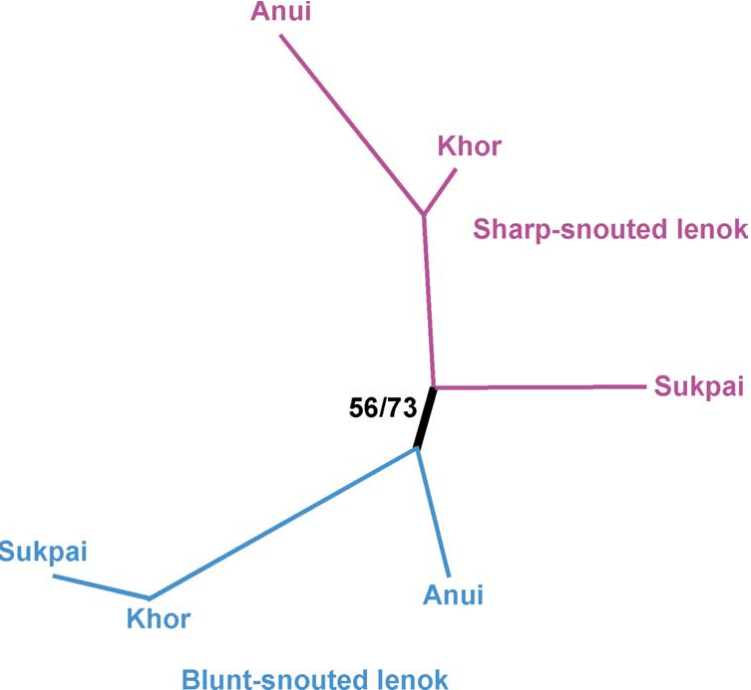
**Population tree based on microsatellites**. Neighbor-joining population tree based on microsatellite data. Bootstrap values for *D*_AS _distances are shown on the left and for Nei's distances on the right.

Microsatellite loci revealed significant deviations from HWE within populations across loci (primarily positive *F*_IS _values), most likely due to the sampling of spawning and post-spawning aggregates of individuals. A test of deviation from HWE across all loci and populations was also significant. The population tree reveals two groups (sharp- and blunt-snouted forms) supported by a moderate *D*_*AS *_bootstrap value (73%) and a lower value (56%) for Nei's genetic distance (Figure 8).

The Bayesian population structure analysis for the entire data set resulted in the highest delta log-likelihood between K = 1 and K = 2, representing the two groups of individuals corresponding to sharp- and blunt-snouted lenoks. Strong structure was further revealed within forms (data not shown), especially for blunt-snouted lenok corresponding to different sampling dates, providing the most plausible cause of deviations from HWE. That is, a Wahlund effect is assumed caused by the pooling of distinct population units into single tests for HWE.

We further tested for introgression between forms by running the Bayesian structure analysis on each sympatric sampling site individually, with K set at 2. Using this analysis there was little to no signal for a mixed ancestory for individual genotypes (Additional File 9).

### Correlation between morphological and genetic distances

Genetic (mtDNA) and morphological distances among populations were highly correlated in sharp-snouted lenok (Mantel's r = 0.75, *P *< 0.001) but weakly correlated in blunt-snouted lenok (Mantel's r = 0.36, *P *= 0.014). Morphological analysis corresponds with genetic data as populations are almost exclusively grouped according to major river basins, except for divergence within the Selenga basin, a phenomenon common to the genetic data set (Figure 3).

## Discussion

### Phylogenetic relationships and taxonomic inferences

Extensive mtDNA screening and comprehensive morphological analysis of *Brachymystax *throughout its Siberian range support that the two forms represent two independent evolutionary lineages, and analysis of 11 microsatellite loci in three sympatric populations (*N *= 130) revealed no signs of introgression. Thus, the hypothesis of "multiple hybrid speciation" [22] is not supported with our selection of genetic markers.

The complete concordance between morphology and mtDNA haplotype as well as no evidence of admixture in sympatric populations analyzed with microsatellite loci strongly suggests that introgression between the two forms is low or non-existent. Nonetheless, hybrids exist [[24-26], Additional File 1], some evidence of introgression has been reported [28] and in captivity, F_1 _hybrids and their progeny (backcrosses and F2) are viable [Alekseev S., unpublished data]. Thus, one or more mechanisms must be operating to prevent higher levels of introgression, such as selection against hybrids in nature. However, a pre-zygotic barrier, such as spatial segregation of spawning may be the primary isolating mechanism. Despite widespread co-occurrence in the Amur [26] and Lena basins [25] blunt-snouted lenok are reported to prefer cooler water during summer months and use distinct spawning grounds. Blunt-snouted lenok spawned both in mountain tributaries (at 3–4°C) and in the main channel (at 5–7°C) of a second-order Amur tributary whereas sharp-snouted lenok spawned in the main channel only, typically in its more downstream reaches [29].

The lack of correlation between genetic and morphological distances in blunt-snouted lenok may be due to the fact that they exist in smaller and more fragmented populations, which would result in higher genetic drift and more stochastic evolutionary change. Likewise, strong genetic drift among basins, as well as fragmentation within basins could explain the range-wide incongruities in allozyme allele distributions between the two forms [26,27,30], and thus more complex models of hybridization and introgression need not be invoked.

The data from China and Korea are difficult to incorporate into our conclusions, although these populations may prove pivotal for understanding the evolutionary history of the genus. The divergent mtDNA lineage in the Yellow River basin for example, is only weakly supported as a sister group to the sharp-snouted clade (data not shown). Lenok from this region are known as *B. lenok tsinlingensis *[31], but [32] and [33] place them together with Siberian blunt-snouted lenoks based on external morphology. [32] assigned the name *B. lenok *to the sharp-snouted form, and *B. savinovi *to the blunt-snouted, but the latter name was shown to be invalid [34]. Alternatively the name *B. tumensis *[35] first used for Tumen River (Korea) lenok is currently used for the blunt-snouted lenok [36]. If the Yellow River and Korean lineage can be definitively defined as blunt-snouted lenok, then the strong correspondence between genetic lineage and form presented in our work would be eroded.

The taxonomy of sharp- and blunt-snouted lenok remains unresolved. The genetic proximity of the Tumen River haplotype to the sharp-snouted lineage (Figure 5) casts serious doubt on the applicability of the name *B. tumensis *for blunt-snouted lenok. Thus, taxonomic harmonization of *Brachymystax *can only be achieved when both genetic and morphological (especially craniological) characters of Chinese and Korean lenoks are compared to material from the Siberian range.

### Comparative phylogeography of blunt- and sharp-snouted lenok

Both lenok forms revealed strong phylogeographic structure with some common patterns such as the existence of highly divergent mtDNA lineages below 56° N and no such variation above 56° N. This suggests that extensive regions of contemporary lenok habitat in Siberia may have been uninhabitable during one or more Pleistocene glacial maxima. In contrast, high within or among basin diversity in more southern latitudes supports the existence of long-term refugia. Interestingly, there has apparently been a complex history within several of these refugia, analogous to the concept of "refugia-within-refugia" promoted for the high lineage diversity of numerous taxa within the Iberian Peninsula in Europe [37]. This "within-refugia" pattern can be seen for the Ob and Primor'e basin blunt-snouted lenoks as well as the Enisey sharp-snouted lenoks. Only the Ob sharp-snouted lenoks run counter to this pattern, as only a single haplotype has been thus far found.

Furthermore, as the most divergent groups within each form are not geographically congruent, different regions apparently served as refugia for each form during unfavorable paleohydrological conditions. For example, relatively divergent haplotypes in sharp-snouted lenok are found in the Selenga/Baikal portion of the Enisei basin where blunt-snouted lenok are wholly absent. Additionally, in contrast to the broadly distributed sharp-snouted lenok, blunt-snouted lenok above 56° N are only found in the Lena basin, a very large system where at least its upstream regions are known to have served as a refuge for an endemic lineage of grayling *Thymallus *[16]. These patterns imply that the two lenok forms, perhaps related to their spatial or temperature preferences differ in both dispersal ability and response to climatic change.

Whereas the majority of genetic variance in both forms was distributed among major basins more specific mechanisms generating phylogeographic structure were inferred. Both long distance dispersal (Uda) and contiguous range expansion (Shantar Islands) underscore the importance of the Amur region as a center of radiation for blunt-snouted lenok. Additionally, the morphological phenogram groups blunt-snouted lenoks from both islands together with those from the Amur. That blunt-snouted lenoks from the Tugur carry the most widespread Amur haplotype, suggests that this river was a corridor between the Amur and the Shantar islands. This supports earlier conclusions of [38] and [39] and is concordant with geological data indicating that Shantar and Sakhalin islands were connected to the continent during glacial maxima. As both genetic and morphological divergences are greater between the Uda and Amur, than the Amur and islands suggests that the Uda may have been colonized during an earlier marine regression than the islands, which were presumably colonized during the LGM. This perspective was supported by the mismatch analysis, revealing expansion from the Amur basin. The age estimate of this event clearly reaches back into the mid-Pleistocene (Figure 7), based on moderate substitution rates of 1%/MY for the CR, or moderately faster rates for the ND-1. The earliest split within blunt-snouted lenok, however, is clearly between populations of the Primor'e region and all others. Coupled with their morphological distinctiveness, Primor'e region blunts have clearly experienced a long isolation from the Amur basin.

The demographic history of sharp-snouted lenoks is more difficult to describe. Mismatch analysis supports exponential growth for the Lena, Amur and Enisei data combined, and long distance dispersal was documented from the Lena to the Amur drainage, as well as continuous range expansion within the Amur and into the Enisei basin. The allopatric fragmentation in sharp-snouted lenoks involving the Selenga-Baikal lineage may correspond to the prior isolation of the Baikal basin and subsequent connection to the Enisei basin as discussed in relation to *Thymallus *[11], or about 500,000 years ago according to geological data [40]. Yet longer periods of isolation are clear for sharp-snouted lenok in the extreme east (Uda) and west (Ob).

In both forms, the CR network reveals haplotypes shared between Lena and Amur basins, as previously described [20] and thus supporting paleohydrological exchange between these major basins. Interestingly, this basin sharing is seen with the ND1 gene in sharp-snouted but not blunt-snouted lenok perhaps reflecting a longer period of allopatry between the two basins for blunts, allowing sufficient time for mutations to occur.

While data from China and Korea adds some confusion to phylogeographic summary of Siberia, a scenario suggesting that the Chinese portion of the lenok's range was colonized by expansion from Siberia southwards [41] is unlikely, or at best oversimplified. Chinese lenok populations contain diverse lineages, perhaps reflecting both long-term relictism as well as admixture from southward expansion.

### Demographic history

The primary demographic inference was that sudden expansions in both forms relate to paleo-climatic conditions much older than the LGM, and thus are better reflected in the more slowly evolving CR. Moreover, signals of sudden expansions are limited to the Amur, Lena, or Enisei, and were not evident in the extreme eastern or western areas of occurrence. These results appear incongruent with countercurrent dispersal [23], as no signal of expansion from Western or Central Siberia can be seen for sharp-snouted lenoks, but rather from the aggregated distributions across major basins. Nonetheless, the occurrence of highly divergent lineages in the east for blunts and the west for sharps supports long-term residence, but there is a lack of genetic resolution for the deep past, so no inference concerning origins of either lineage can be made.

### Paleohydrological dynamics

Although major basins account for a substantial percentage of both morphological and genetic variation, underscoring isolation, the inferred trans-basin expansions support repeated availability of paleohydrological dispersal (active or passive) corridors. This implies a dynamic paleo-landscape with refugia perhaps in the form of periglacial lakes and shifting drainage patterns stemming from headwater captures and river flow reversals. More specific details concerning Siberia's paleohistory remain highly controversial, and can not be addressed based on the moleulcar phylogeography of *Brachymystax *alone. Glacial reconstructions range from nearly complete coverage of northern Siberia by a marine-based ice-sheet [13,42,43] to individual ice caps centered on arctic archipelagos that advanced onto adjacent shelves [44]. It remains to be seen whether or not phylogeographic models, and especially comparative phylogeography could be used to support or refute one of these contrasting scenarios of paleo-Siberian landscapes. While prior studies [11,16,20,21] have invoked highy dynamic paleo-climatic events to explain the distribution of salmonid mtDNA lineages in various Siberian basins, we offer no further speculations on these scenarios. It is clear, however, that the level of resolution in understanding the effect of paleohydrology on current genetic diversity and distribution of freshwater fishes in Siberia lags behind that of Europe and North America.

## Conclusion

To date, there have been no Siberian-wide phylogeographic studies of aquatic organisms, and as well, an underestimation of the effects of paleo-hydrological dynamics on their current distributions. Our data clearly identify a "northern" gradient in lineage diversity particularly evident across 56° N, multiple genetic imprints of inter-basin exchange, long-distance dispersal and sudden expansions across broad expanses of Siberia in two evolutionarily independent lineages of the salmonid fish *Brachymystax lenok*. Genetic signals for these events clearly date to climatic periods prior to the LGM, and thus were better reflected by the more slowly evolving mtDNA control region rather than the faster mutating NADH-1 gene segment.

Several previous hypotheses concerning the evolution of lenok are not supported by our data. Namely, multiple hybrid speciation [22], countercurrent dispersal [23], and a Siberian source for lenok in the Chinese portion of their current range [41]. While no inference can be drawn on the potential origins of the two forms of lenok, both exhibit highly divergent lineages located at opposite end's of the composite distribution range (to the extreme west for the sharp-snouted form, and the extreme east for the blunt-snouted form). A simple taxonomic division of two genetic lineages of lenok, corresponding to two phenotypically recognizable forms is compromised by the existence of a third mtDNA lineage in China and Korea where phenotypic data is lacking. Our data support species recognition of the two forms, but taxonomic harmonization rests on the ability to integrate both genetic and phenotypic data from the China and Korea.

## Methods

### Sampling

Blunt-snouted (n = 663) and sharp-snouted (n = 1028) lenoks from 78 locations were sampled in 1975–2005. In 22 locations both forms were collected, whereas 23 sites yielded exclusively blunt-and 33 exclusively sharp-snouted lenoks (Table 1, Figure 1). Several of the most closely related taxa were added for comparative purposes to the genetic analyses including one *Hucho hucho*, one *Hucho taimen*, and one Sakhalin taimen *Parahucho perryi*; and for the morphological analysis 101 *H. taimen *from various basins.

**Table 1:** Site names, basins/regions, sample sizes and coordinates for lenok (*Brachymystax*) samples used in this study

			Number of individuals sampled				Geo_coordinates	
					
		Site	mtDNA		Morphology			
					
Site	Drainage		Sharp-snouted	Blunt-snouted	Sharp-snouted	Blunt-snouted	Latitude	Longitude
**Ob basin**								
L. Markakol'	Kal'dzhir→Chernyi Irtysh→Zaisan→Irtysh→Ob→Kara Sea	1	4		60		48°44'	85°45'
R. Kal'dzhir	Chernyi Irtysh→Zaisan→Irtysh→Ob→Kara Sea	2	2		93		48°36'	85°10'
R. Kara-Kaba	Chernyi Irtysh→Zaisan→Irtysh→Ob→Kara Sea	3	2				48°53'	86°10'
R. Pyzha	Biya→Ob→Kara Sea	4		2		34	51°46'	87°06'
R. Biya	Ob→Kara Sea	5		3		3	51°49'	87°09'
R. Mrassu	Tom→Ob→Kara Sea	6		1			52°43'	88°35'
R. Kabyrza	Mrassu→Tom→Ob→Kara Sea	7		1			52°52'	88°52'
R. Bol'shoi Kemchug	Kemchug→Chulym→Ob→Kara Sea	8		2		6	55°49'	91°34'
**Enisei Basin (Selenga River)**								
R. Orkhon	Selenga→Baikal→Enisei→Kara Sea	9	2				49°20'	105°30'
R. Ero	Orkhon→Selenga→Baikal→Enisei→Kara Sea	10	2				49°05'	107°14'
R. Ider	Selenga→Baikal→Enisei→Kara Sea	11			20		48°44'	98°15'
R. Delger-Muren	Selenga→Baikal→Enisei→Kara Sea	12			10		49°32'	99°13'
L. Chovsgol	Selenga→Baikal→Enisei→Kara Sea	13	1		15		51°10'	100°35'
R. Khankhgol	Chovsgol→Selenga→Baikal→Enisei→Kara Sea	14	2				51°26'	100°41'
**Enisei Basin (Lake Baikal)**								
R. Frolikha	Baikal→Enisei→Kara Sea	15	2				55°31'	109°53'
R. Shegnanda	Baikal→Enisei→Kara Sea	16			23		54°58'	109°32'
R. Bol'shaya	Baikal→Enisei→Kara Sea	17			5		54°28'	109°30'
**Enisei Basin**								
R. Kyzyl-Khem	Malyi Enisei→Enisei→Kara Sea	18	1				51°30'	97°57'
R. Kazyr	Tuba→Enisei→Kara Sea	19			35		53°42'	94°06'
R. Verkhnyaya Surnikha	Enisei→Kara Sea	20			6		60°05'	90°34'
R. Stolbovaya	Podkamennaya Tunguska→Enisei→Kara Sea	21			22		62°09'	91°25'
R. Varlamovka	Enisei→Kara Sea	22	5		14		62°23'	89°24'
**Lena (upper) basin**								
L. Nomama	Asektamur→Chaya→Lena→Laptev Sea	23	1		14		56°16'	110°16'
L. Amudisa	Kalar→Vitim→Lena→Laptev Sea	24	2		2		56°33'	119°04'
R. Kalakan	Kalar→Vitim→Lena→Laptev Sea	25		1	2	5	56°14'	119°32'
L. Leprinidokan	Kuanda→Vitim→Lena→Laptev Sea	26	2		104	13	56°33'	117°29'
R. Kuanda	Vitim→Lena→Laptev Sea	27	2	4	124	6	56°31'	117°26'
R. Kuanda	Vitim→Lena→Laptev Sea	28			5	18	56°25'	117°23'
R. Kuanda	Vitim→Lena→Laptev Sea	29			8	15	other	sites
Nameless lake	Kuanda→Vitim→Lena→Laptev Sea	30				13	56°25'	117°30'
L. Amalyk	Amalyk→Vitim→Lena→Laptev Sea	31		2		10	57°35'	117°17'
**Lena (middle) basin**								
L. Bol'shoe	Chara→Olekma→Lena→Laptev Sea	32	4		8		56°38'	117°36'
Nameless lake	Itchilyak→Evonokit→Khani→Olekma→Lena→Laptev Sea	33		2		15	57°11'	119°50'
Nameless lake	Itchilyak→Evonokit→Khani→Olekma→Lena→Laptev Sea	34				14	57°10'	119°52'
R. Utuk	Bol. Toko→Mulam→Idyum→Algama→Uchur→Aldan→Lena→Laptev Sea	35	2		5		55°55'	130°45'
L. Bol'shoe Toko	Mulam→Idyum→Algama→Uchur→Aldan→Lena→Laptev Sea	36	3		71		56°02'	130°53'
R. Yudoma	Maya→Aldan→Lena→Laptev Sea	37	3				61°11'	140°33'
R. Kele	Aldan→Lena→Laptev Sea	38	4	4			63°25'	130°27'
R. Vilui	Lena→Laptev Sea	39	2		8		65°32'	106°43'
R. Morkoka	Markha→Vilui→Lena→Laptev Sea	40	3		86		64°36'	112°29'
R. Tuyng	Vilui→Lena→Laptev Sea	41	2				63°49'	121°27'
**Lena (lower) basin**								
R. Dyanyshka	Lena→Laptev Sea	42	1	2	6	21	65°27'	126°56'
R. Kundudei	Lena→Laptev Sea	43	1	2			65°47'	125°34'
R. Undyulyung	Lena→Laptev Sea	44	3		20	8	66°16'	123°58'
R. Tirekhtyakh	Undulung→Lena→Laptev Sea	45	4		10	7	66°13'	124°42'
R. Sobolokh-Mayan	Lena→Laptev Sea	46	2		5		67°14'	123°41'
**Indigirka basin**								
R. Nizhnyi Labynkyr	Tuora-Yuryakh→Indigirka→East Siberian Sea	47			21		62°36'	143°36'
R. Indigirka	East Siberian Sea	48	3				66°36'	143°00'
**Kolyma basin**								
R. Krivaya	Omolon→Kolyma→East Siberian Sea	49	1		36		64°38'	160°45'
R. Popovka	Kolyma→East Siberian Sea	50	4		10		65°12'	151°39'
**Amur basin**								
R. Onon	Shilka→Amur→Sea of Okhotsk	51	2				48°35'	110°48'
R. Tok	Zeya→Amur→Sea of Okhotsk	52		2		43	55°38'	130°01'
R. Bureya		53	1	1			51°38'	133°24'
R. Levaya Bureya	Bureya→Amur→Sea of Okhotsk	54		**2**			51°55'	134°53'
R. Gobili	Anui→Amur→Sea of Okhotsk	55	1	1	12	8	49°15'	138°19'
R. Ertukuli	Anui→Amur→Sea of Okhotsk	56	1				49°18'	138°03'
R. Anui	Amur→Sea of Okhotsk	57	2	2			49°17'	137°55'
R. Anui	Amur→Sea of Okhotsk	58			19	49	49°14'	137°01'
R. Manoma	Anui→Amur→Sea of Okhotsk	59	3	3			49°21'	137°24'
R. Sukpai	Khor →Ussuri→Amur→Sea of Okhotsk	60			11	15	47°45'	137°18'
R. Khor	Ussuri→Amur→Sea of Okhotsk	61	3	5	31	50	47°38'	135°52'
R. Suluk	Amgun'→Amur→Sea of Okhotsk	62		2			51°05'	134°06'
R. Merek	Amgun'→Amur→Sea of Okhotsk	63		2			51°17'	134°47'
R. Duki	Amgun'→Amur→Sea of Okhotsk	64	1		49	29	51°28'	135°46'
R. Im	Amgun'→Amur→Sea of Okhotsk	65		2			52°30'	138°14'
**Sakhalin Island**								
R. Bol'shoi Vagis	Sea of Okhotsk (Amur Liman)	66		5			52°30'	142°00'
R. Ten'gi	Sea of Okhotsk (Amur Liman)	67				6	52°44'	142°03'
R. Pyrki	Sea of Okhotsk (Amur Liman)	68				7	52°52'	142°05'
**Bol'shoi Shantar Island**								
R. Yakshina	Sea of Okhotsk	69		1		50	54°55'	137°32'
R. Bol'shoi Anaur	Sea of Okhotsk	70		4			54°47'	137°40'
**Tugur basin**								
R. Konin	Tugur→Sea of Okhotsk	71	3	2			53°14'	136°06'
**Uda basin**								
R. Uda	Sea of Okhotsk	72			8	30	54°08'	131°51'
R. Uda	Sea of Okhotsk	73	1	1	10	6	54°33'	134°26'
Popkovskie lakes	Uda→Sea of Okhotsk	74	1	4	4	47	54°39'	135°10'
L. Urgos	Uda→Sea of Okhotsk	75				35	54°39'	135°15'
**Primor'e (Sea of Japan basin)**								
R. Edinka	Sea of Japan	76		1		7	47°12'	138°37'
R. Samarga	Sea of Japan	77				38	47°17'	138°39'
R. Beya	Serebryanka→Sea of Japan	78		1		22	45°02'	136°34'
**TOTAL**			**91**	**67**	**992**	**630**		

### Amplification and sequencing

Whole genomic DNA was extracted using a standard high-salt protocol. Two mtDNA fragments, the control region (CR) and NADH-1 subunit (ND1) were amplified using the polymerase chain reaction (PCR). The complete CR (including segments of flanking tRNA) was amplified in 97 *B. lenok *and three outgroup individuals using the primers LRBT-25 and LRBT-1195 (8). The remaining CR sequences were taken from previously published research (GenBank accession no. AY230451–AY230472). The ND1 primers BlNDF and BlNDR [45] were used to amplify 142 *B. lenok *as well as outgroup individuals. PCR conditions (25 μl reactions) were identical to those described in [45], and sequences of the left-domain of the CR (486 bp) and complete ND1 (987 bp) were produced on an ABI-3100 genotyper. When necessary, primers designed to amplify shorter segments were used to amplify degraded DNA (see Additional File 10). New mtDNA sequences have been deposited under accession numbers [GenBank: EU395714–EU395769].

### Sequence alignment and phylogenetic analysis

All sequences were easily aligned by eye including an alignment incorporating one sequence from South Korea (GenBank accession no. AF125519), and 14 from China [41]. Quantitative assessment was limited to the sequences produced in our laboratory.

Sequences were imported into PAUP*4.0b10 [46] for phylogenetic analyses, pairwise sequence divergence (uncorrected *p *distances) and the number of transitions and transversions. Saturation in ND1 was assessed by plotting the number of transitions and transversions against uncorrected *p *distances for each codon position. A chi-square (χ^2^) test was used to evaluated base composition homogeneity in the ND1 gene for each codon position.

To evaluate relationships among closely related haplotypes, unrooted networks were constructed with a 95% parsimony criterion using TCS ver 1.13 [47]. Between-group variation was calculated using net nucleotide divergence (*Da*) in MEGA version 2.1 [48]. Haplotype or clade divergence was also calculated using *Da *distances between groups using the Kimura two-parameter model. Uncorrected *p *distances were used for divergence estimates between in- and outgroup taxa.

Maximum parsimony (MP), Maximum-Likelihood (ML) and Neighbor-Joining (NJ) were used for phylogenetic reconstruction. Modeltest 3.0 [49] was used to choose models of nucleotide evolution. To estimate the most likely topology for ML and MP methodologies, heuristic searches (10 replicates) started with stepwise addition trees, with each replicate beginning with a random order of sequences. Bootstrap analysis was used to estimate node support with 10000 (NJ and MP) or 1000 replicates (ML). Full heuristic search algorithms were applied for the MP and the "fast" stepwise addition method for the ML analysis.

### Amova

Genetic variation among and within major basins was evaluated with an analysis of molecular variance (AMOVA) using Arlequin 3.1 [50]. The AMOVA structure was defined by major ocean basins (Arctic/Pacific), then by large river basins or regions (Ob, Enisei, Lena including Indigirka and Kolyma, Amur, Islands, Primor'e, and Uda and Tugur) (see Table 1). A more detailed overview of within and among basin differentiation is provided with a table of average (and corrected) pairwise differences also done with Arlequin.

### Mismatch Analysis

The demographic signature of mtDNA haplotype variation was evaluated with the pairwise mismatch distribution [51]. The goodness-of-fit of the observed data to a simulated model of expansion was tested with the sum of squared deviations and Harpending's raggedness index [52]. The age of expansion was estimated with the formula τ = 2 μ*t*, where τ is drawn from the mismatch distribution, μ equals the aggregate substitution rate across all nucleotides per generation (5 years for lenok) and *t *is the expansion time in generations, graphically displayed in years. The aggregate substituion rate was based on a plausible range of divergence rates for mtDNA in salmonid fishes [see Discussions in [8,11,20,53] and references therin]. Thus, estimates of the age of expansion were plotted across a range of substitution rates, whereby it is assumed that the substitution rate of the coding ND1 gene is higher than the control region, which is common for salmonid fishes [45,54], and supported by our haplotype diversities (i.e., twice the number of haplotypes for the ND-1 gene compared to the CR). A Kolmogorov-Smirnov two-sample test was used to test the distribution of observed values against those expected under the null hypothesis of a stable population using SPSS ver. 12.0.

### Microsatellites

To assess introgression between sharp- and blunt-snouted lenoks, we applied bi-parentally inherited microsatellites to three sets of sympatric populations from the Amur basin: the Anui (n = 56), Khor (n = 48), and Sukpai (n = 26) rivers.

Four tri-nucleotide and three tetra-nucleotide microsatellite loci [55], and four unpublished di-nucleotide microsatellite loci were analyzed (see Supplementary Materials). All forward primers were fluorescently labeled and PCR and genotyping were performed as described in [55], (see Additional File 10). Exact probability tests for deviations from Hardy-Weinberg equilibrium (HWE) across populations (within loci) and loci (within populations), exact tests for deviations from genotypic linkage equilibrium (LE) across populations, and tests for genic differentiation among populations were performed with Genepop 3.2a [56]. Corrections for multiple significance tests were performed using a sequential Bonferroni-type correction [57].

To estimate the proportion of each individual's genome originating in each parental species and the patterns of intraspecific variability among sharp- and blunt snouted lenoks we used the Structure [58]. This software implements a Bayesian model-based clustering algorithm that reveals population structure in a data set by resolving clusters of individuals that minimize Hardy-Weinberg and linkage disequilibrium. The parameter settings included the assumption of admixture and a correlated allele frequencies model. In exploratory runs we did not provide the software with prior information regarding the origin of individuals, but in final runs, and when analyzing the three sympatric populations separately, individuals from the two forms were assumed to represent pure blunt- or sharp-snouted lenoks, and used as a proxy for determining the degree of admixture of the individuals within each sympatric population. Structure was run for 100,000 steps, of which the first 10,000 where discarded as burn-in and we conducted five independent replicates of the MCMC for each value of k. We also performed analyses using the independent allele frequencies model to test for robustness of our conclusions to the violation of prior assumptions because of recent suggestions that the choice of the model might strongly influence the outcome of the clustering algorithm [59,60].

### Morphological analysis

External morphology was analyzed using fresh fish or heads fixed with salt. Measurements were taken with dividers to the nearest mm. We used a modified morphometric scheme [61] for salmonids [see [62]], with reductions. Osteological characters were assessed according to [63] (for details see [25]). To adequately reflect shape variation, body measurements were expressed as percent of fork length (FL), head measurements as percent of head length (c), skull measurements as percent of skull base length (Lcr) and bone widths (depths) as percent of bone lengths. Allometric growth is known to be weak in lenok above 20 cm FL. Therefore, to reduce potential allometric effects only fish larger than 25 cm FL were used. A total of 38 indexes and 8 counts were analyzed (see Additional File 10).

To explore the relationship in morphology between forms and among populations within forms both principal component analysis (PCA) and cluster analysis were done using the NTSYS-pc package, v2.0 [64]. For PCA, indexes and counts of individual fish were used. Eigenvectors were calculated from the variance-covariance matrix and the eigenvector loadings were scaled so that the length of the vectors equaled the square root of their eigenvalues. For cluster analysis, index and count means were standardized and used to calculate taxonomic distances between populations. These distances served as raw data for construction of a UPGMA phenogram. To further control for potential allometric effects, the first factor in the PCA was regressed with body size.

To quantify the morphological differentiation between forms we used a canonical discriminant analysis (CDA), using the first three PCA factors as input variables, and an equal probability of assignment of each individual to a form (blunt or sharp). A discriminant function (DF) was derived from a 25% random sample of the data set, and this DF was then applied to assign individuals to a form.

### Comparison of morphological and genetic data

We assessed the correlation between genetic and morphological data across the range of the two lenok forms using Mantel tests, done in the NTSYS-pc package. To produce a pairwise genetic (mtDNA) distance matrix, between group variation (corrected for within-group variation) was calculated using the net nucleotide distances (D_A_) (Kimura two-parameter model). For the morphological distance matrix, the same taxonomic distances generated for the clustering were used. As not all populations had matching genetic and morphological matrices, only 22 populations of sharp-snouted, and 17 populations of blunt-snouted lenoks were used. The significance of Mantel's r was evaluated with 9999 permutations.

## Authors' contributions

EF, SA, PA, and SW participated in the design and coordination of the project. EF and SW carried out the molecular genetic studies whereas SA carried out the morphological work and collected most of the samples. SW wrote the manuscript and all authors read, revised and approved the final version.

## Supplementary Material

Additional File 1 Figure 4a from [25] showing the intermediate position of hybrids between pure sharp-snouted and blunt-snouted lenok from the Kuanda River basin. Hybrids were first identified "by eye" in the field but corroborated with this multivariate analysis (PCA) based on 37 morphological characters. Additionally, a subset of these hybrid individuals were screened for allozyme variation and shown to be heterozygous at loci diagnostic for the two forms in that basin [26]. Click here for file

Additional File 2 Scatterplot demonstrating no relation between PCA factor 1 and fish size (given as fork length). Click here for file

Additional File 3 List of haplotypes and their frequencies for the control region gene analyzed across the populations sampled (N = 150 *B. lenok *individuals). Click here for file

Additional File 4 List of haplotypes and their frequencies for the ND1 gene analyzed across the populations sampled (N = 139 *B. lenok *individuals). Click here for file

Additional File 5 Pairwise haplotype differences among six basins for both forms of lenok. The upper diagonal represents average pairwise differences, the diagonal within basin differences, and the lower diagonal pairwise differences corrected for within basin variation. Click here for file

Additional File 6 Results of the AMOVA of pairwise haplotype differences with the structure defined by major ocean basins, and river drainages (or regions when considering Islands of the Okhostsk Sea) within basins. Signficance fo the variance components is based on 1000 permutations. Click here for file

Additional File 7 The CR pairwise mismatch distribution for (A) – blunt-snouted lenok; (B) – sharp-snouted lenok; and (C) – sharp-snouted lenok after removal of regionally restricted haplotypes. Click here for file

Additional File 8 The ND1 pairwise mismatch distribution for (A) – blunt-snouted lenok; (B) – blunt-snouted after removal of regionally restricted haplotypes; (C) – sharp-snouted lenok; and (D) – sharp-snouted after removal of regionally restricted haplotypes. Click here for file

Additional File 9 Graphical display of ancestory coefficients from the Bayesian simulations from the program Structure. Simulations were run with the assumption that K = 2 (derived from prior simulations that are not shown), correlated allele frequencies, and an admixture model. Five replicates for each basin are shown. Click here for file

Additional File 10 Details for amplification, sequencing, primers and morphological measurements used in this study. Click here for file

Additional File 11 The identical tree (black/white) as shown in Figure 3, but now including the site numbers as listed in table 1. Click here for file

Additional File 12 Extended list of acknowledgements. Click here for file
