# Supplementary figures and images for: The evolutionary history of sharp- and blunt-snouted lenok (Brachymystax lenok (Pallas, 1773)) and its implications for the paleo-hydrological history of Siberia
Source: BMC Evol Biol. 2008 Feb 6;8:40. doi: 10.1186/1471-2148-8-40 (PMC2275220; doi:10.1186/1471-2148-8-40)

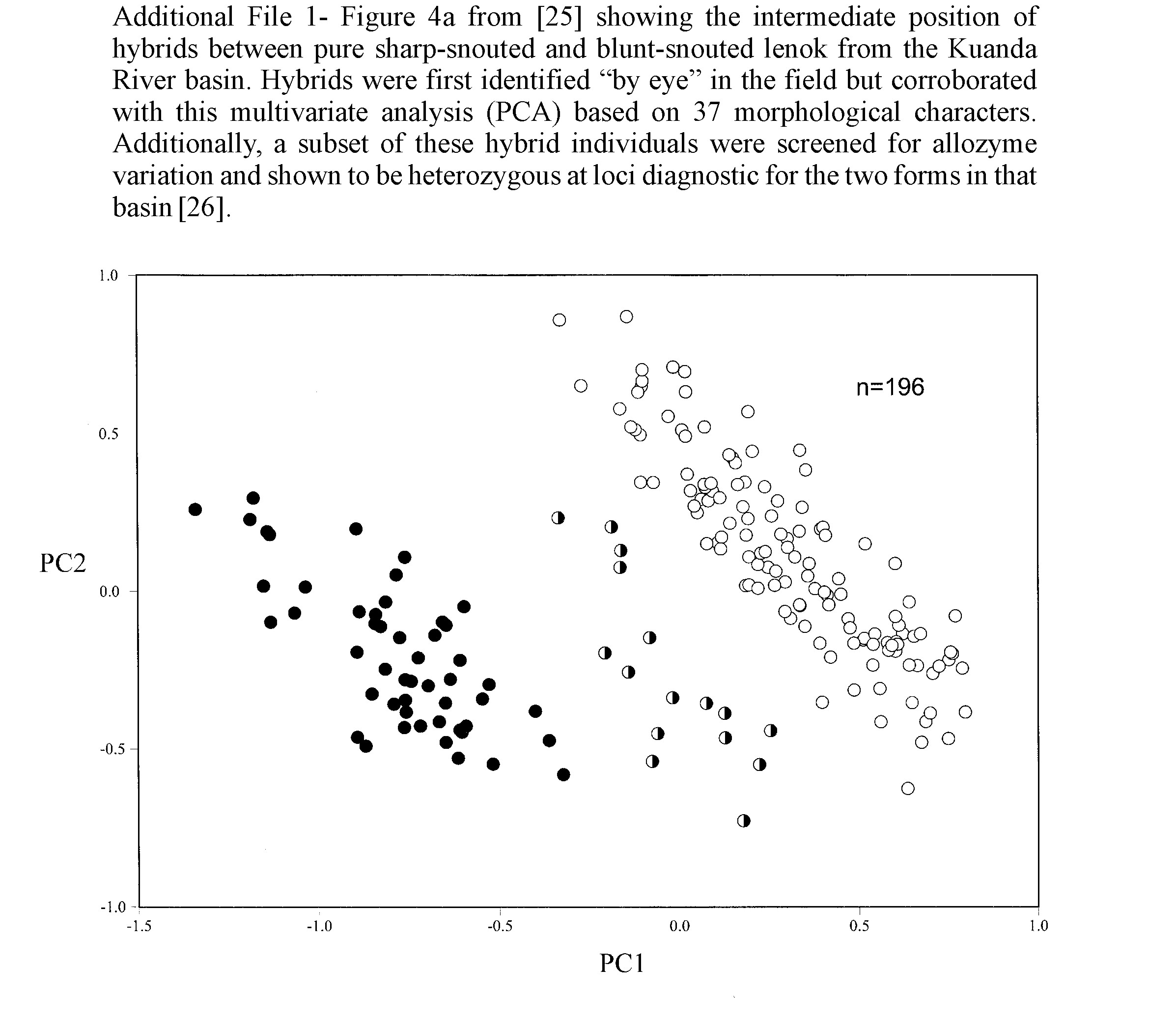

Supplement: Additional File 1 — Figure 4a from [25] showing the intermediate position of hybrids between pure sharp-snouted and blunt-snouted lenok from the Kuanda River basin. Hybrids were first identified "by eye" in the field but corroborated with this multivariate analysis (PCA) based on 37 morphological characters. Additionally, a subset of these hybrid individuals were screened for allozyme variation and shown to be heterozygous at loci diagnostic for the two forms in that basin [26]. [file 1471-2148-8-40-S1.JPEG]

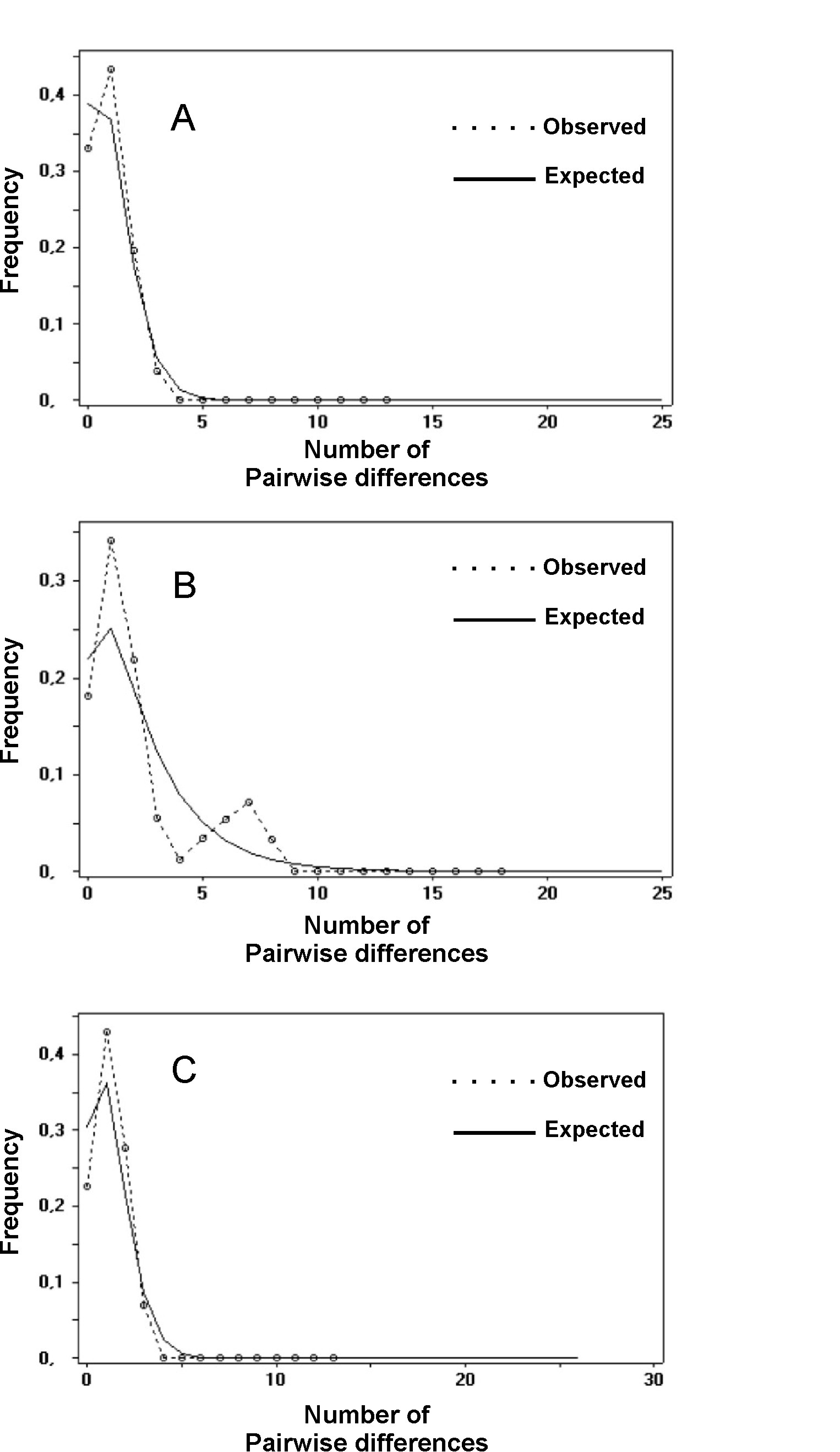

Supplement: Additional File 5 — Pairwise haplotype differences among six basins for both forms of lenok. The upper diagonal represents average pairwise differences, the diagonal within basin differences, and the lower diagonal pairwise differences corrected for within basin variation. [file 1471-2148-8-40-S5.jpeg]

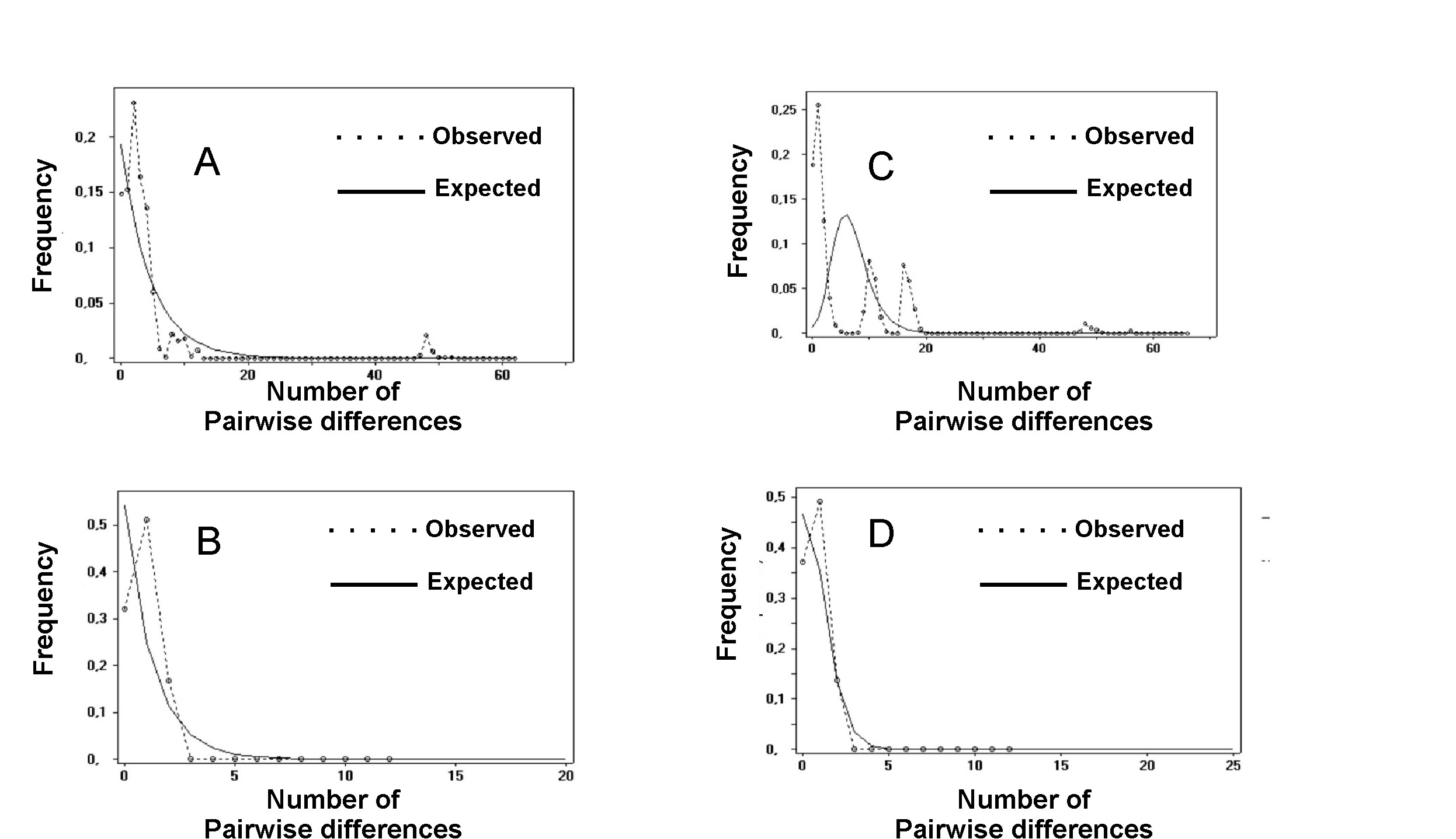

Supplement: Additional File 6 — Results of the AMOVA of pairwise haplotype differences with the structure defined by major ocean basins, and river drainages (or regions when considering Islands of the Okhostsk Sea) within basins. Signficance fo the variance components is based on 1000 permutations. [file 1471-2148-8-40-S6.JPEG]

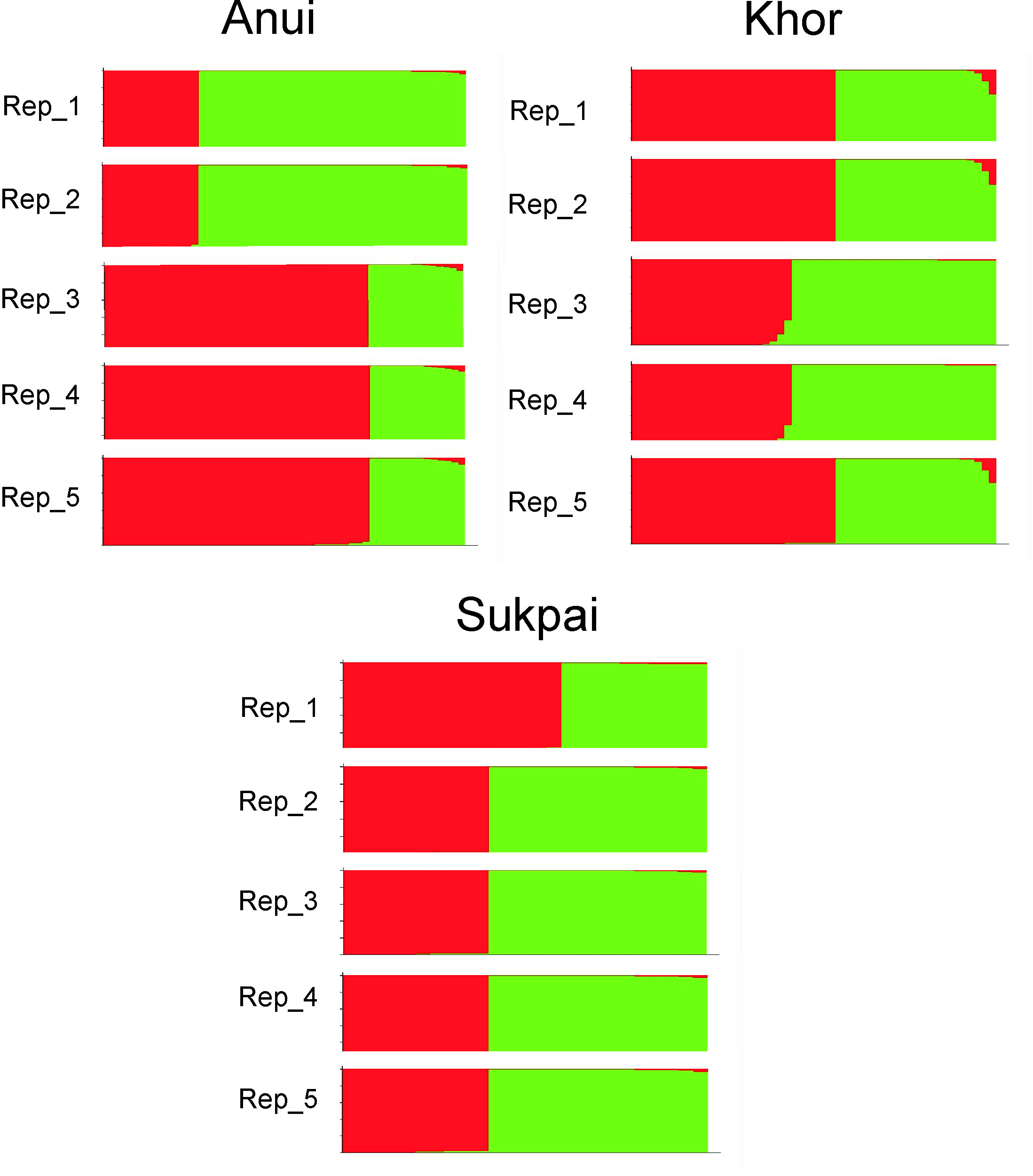

Supplement: Additional File 8 — The ND1 pairwise mismatch distribution for (A) – blunt-snouted lenok; (B) – blunt-snouted after removal of regionally restricted haplotypes; (C) – sharp-snouted lenok; and (D) – sharp-snouted after removal of regionally restricted haplotypes. [file 1471-2148-8-40-S8.JPEG]

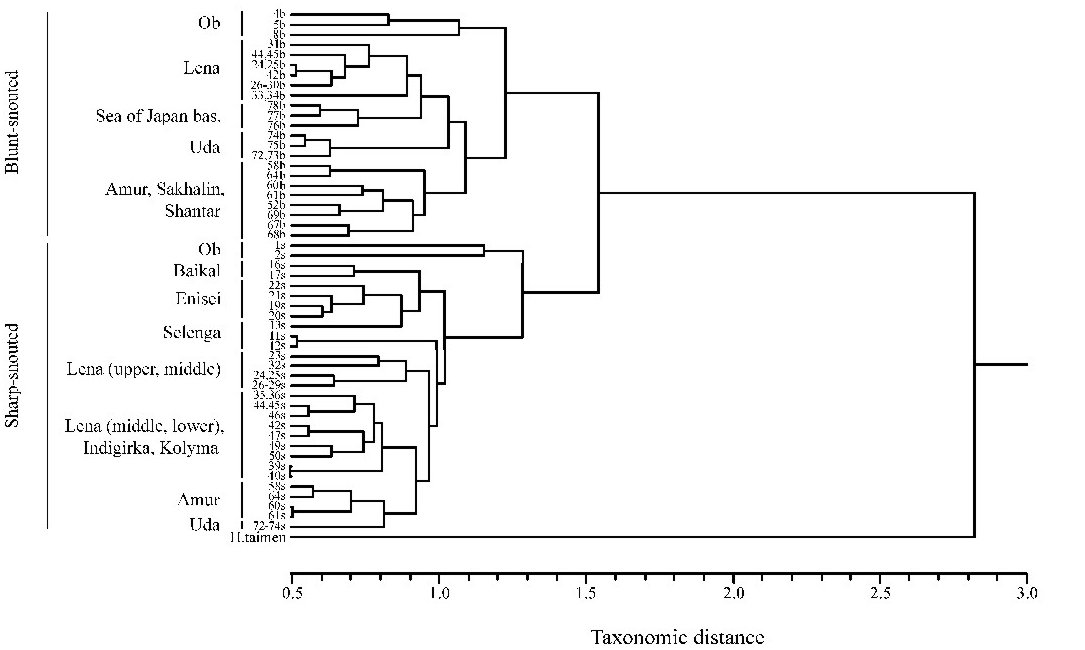

Supplement: Additional File 11 — The identical tree (black/white) as shown in Figure 3, but now including the site numbers as listed in table 1. [file 1471-2148-8-40-S11.JPEG]
