# Supplementary material for: The evolutionary history of sharp- and blunt-snouted lenok (Brachymystax lenok (Pallas, 1773)) and its implications for the paleo-hydrological history of Siberia
Source: BMC Evol Biol. 2008 Feb 6;8:40. doi: 10.1186/1471-2148-8-40 (PMC2275220; doi:10.1186/1471-2148-8-40)
Supplement: Additional File 2 — Scatterplot demonstrating no relation between PCA factor 1 and fish size (given as fork length). [file 1471-2148-8-40-S2.DOC]

**Additional File 2. Scatterplots demonstrating that there was**

**no relation between PCA factor 1 and fish size (given as fork**

**length) for both sharp- and blunt-snouted lenok.**

**Sharp-snouted lenok**

y = 0,0003x + 0,3142

R

2

= 0,0054

-0,6

-0,4

-0,2

0

0,2

0,4

0,6

0,8

1

0

100

200

300

400

500

600

700

**Fork length (mm)**

**PC1**

**Blunt-snouted lenok**

y = -0,0001x - 0,4696

R

2

= 0,0021

-1,2

-1

-0,8

-0,6

-0,4

-0,2

0

0

100

200

300

400

500

600

700

**Fork length (mm)**

**PC1**
