# Supplementary material for: The evolutionary history of sharp- and blunt-snouted lenok (Brachymystax lenok (Pallas, 1773)) and its implications for the paleo-hydrological history of Siberia
Source: BMC Evol Biol. 2008 Feb 6;8:40. doi: 10.1186/1471-2148-8-40 (PMC2275220; doi:10.1186/1471-2148-8-40)
Supplement: Additional File 3 — List of haplotypes and their frequencies for the control region gene analyzed across the populations sampled (N = 150 B. lenok individuals). [file 1471-2148-8-40-S3.DOC]

### Additional File 3 - List of haplotypes and their frequencies for the mtDNA control region (CR) analyzed across populations of blunt- and sharp-snouted lenok (*N* = 150). CR haplotype numbers correspond to those in Figure 5. Site numbers correspond to those in Figure 1.

|  |  | **Control Region (CR) Haplotype Number**  **Blunt-snouted lenok Sharp-snouted lenok** | | | | | | | | | | | | | | | | | | |
| --- | --- | --- | --- | --- | --- | --- | --- | --- | --- | --- | --- | --- | --- | --- | --- | --- | --- | --- | --- | --- |
| **No.** | **Site/Basin** | CR1 | CR2 | CR3 | CR4 | CR5 | CR6 | CR7 | CR8 | CR9 | CR10 | CR11 | CR12 | CR13 | CR14 | CR15 | CR16 | CR17 | CR18 | CR19 |
| **Ob basin** | | | | | | | | | | | | | | | | | | | | |
| 1 | Markakol’ |  |  |  |  |  |  |  |  |  |  |  |  |  |  |  |  |  |  | 4 |
| 2 | Kal’dzhir |  |  |  |  |  |  |  |  |  |  |  |  |  |  |  |  |  | 2 |  |
| 3 | Kara-Kaba |  |  |  |  |  |  |  |  |  |  |  |  |  |  |  |  |  |  | 2 |
| 4 | Pyzha |  |  |  |  |  |  | 2 |  |  |  |  |  |  |  |  |  |  |  |  |
| 5 | Biya |  |  |  |  |  | 3 |  |  |  |  |  |  |  |  |  |  |  |  |  |
| **Enisei (Selenga)** | | | | | | | | | | | | | | | | | | | | |
| 9 | Orkhon |  |  |  |  |  |  |  |  |  |  |  |  |  |  |  | 2 |  |  |  |
| 10 | Ero |  |  |  |  |  |  |  |  |  |  |  |  |  |  |  | 2 |  |  |  |
| 13 | Chovsgol |  |  |  |  |  |  |  |  |  |  |  |  |  |  |  | 1 |  |  |  |
| 14 | Khankhgol |  |  |  |  |  |  |  |  |  |  |  |  |  |  |  | 2 |  |  |  |
| **Enisei (Baikal)** | | | | | | | | | | | | | | | | | | | | |
| 15 | Frolikha |  |  |  |  |  |  |  |  |  |  |  |  |  |  |  | 2 |  |  |  |
| **Enisei** | | | | | | | | | | | | | | | | | | | | |
| 18 | Kyzyl-Khem |  |  |  |  |  |  |  |  |  |  |  |  |  |  | 1 |  |  |  |  |
| 22 | Varlamovka |  |  |  |  |  |  |  |  |  |  |  |  |  |  | 5 |  |  |  |  |
| **Lena (upper)** | | | | | | | | | | | | | | | | | | | | |
| 23 | Nomama |  |  |  |  |  |  |  |  |  |  |  |  |  |  | 1 |  |  |  |  |
| 24 | Amudisa |  |  | 1 |  |  |  |  |  |  |  |  | 2 |  |  |  |  |  |  |  |
| 25 | Kalakan |  |  | 1 |  |  |  |  |  |  |  |  |  |  |  |  |  |  |  |  |
| 26 | Leprindokan |  |  |  |  |  |  |  |  |  |  |  | 2 |  |  |  |  |  |  |  |
| 27 | Kuanda |  |  | 3 |  |  |  |  |  |  |  |  | 2 |  |  |  |  |  |  |  |
| 31 | Amalyk |  |  | 2 |  |  |  |  |  |  |  |  |  |  |  |  |  |  |  |  |
| **Lena (middle)** | | | | | | | | | | | | | | | | | | | | |
| 32 | Bol’shoe Leprindo |  |  |  |  |  |  |  |  |  |  |  |  |  |  | 4 |  |  |  |  |
| 34 | Nameless lake |  |  | 2 |  |  |  |  |  |  |  |  |  |  |  |  |  |  |  |  |
| 35 | Utuk |  |  |  |  |  |  |  |  |  |  |  |  | 2 |  |  |  |  |  |  |
| 36 | Bol’shoe Toko |  |  |  |  |  |  |  |  |  |  |  |  | 3 |  |  |  |  |  |  |
| 37 | Yudoma |  |  |  |  |  |  |  |  |  |  |  |  |  | 1 | 2 |  |  |  |  |
| 38 | Kele |  |  | 4 |  |  |  |  |  |  |  |  |  |  | 2 | 2 |  |  |  |  |
| 39 | Vilui |  |  |  |  |  |  |  |  |  |  |  |  |  | 2 |  |  |  |  |  |
| 40 | Morkoka |  |  |  |  |  |  |  |  |  |  |  |  |  | 3 |  |  |  |  |  |
| 41 | Tuyng |  |  |  |  |  |  |  |  |  |  |  |  |  | 2 |  |  |  |  |  |
| **Lena (lower)** | | | | | | | | | | | | | | | | | | | | |
| 42 | Dyanyshka |  |  | 2 |  |  |  |  |  |  |  |  |  |  | 1 |  |  |  |  |  |
| 43 | Kundudei |  |  | 2 |  |  |  |  |  |  |  |  |  |  | 1 |  |  |  |  |  |
| 44 | Undyulyung |  |  |  |  |  |  |  |  |  |  |  |  |  | 2 |  |  |  |  |  |
| 45 | Tirekhtyakh |  |  |  |  |  |  |  |  |  |  |  |  |  | 4 |  |  |  |  |  |
| 46 | Sobolokh-Mayan |  |  |  |  |  |  |  |  |  |  |  |  |  | 2 |  |  |  |  |  |
| **Indigirka** | | | | | | | | | | | | | | | | | | | | |
| 48 | Indigirka |  |  |  |  |  |  |  |  |  |  |  |  |  | 3 |  |  |  |  |  |
| **Kolyma** | | | | | | | | | | | | | | | | | | | | |
| 49 | Krivaya |  |  |  |  |  |  |  |  |  |  |  |  | 1 |  |  |  |  |  |  |
| 50 | Popovka |  |  |  |  |  |  |  |  |  |  |  |  | 4 |  |  |  |  |  |  |
| **Amur** | | | | | | | | | | | | | | | | | | | | |
| 51 | Onon |  |  |  |  |  |  |  |  |  |  |  |  |  |  | 2 |  |  |  |  |
| 52 | Tok |  |  |  |  |  |  |  | 2 |  |  |  |  |  |  |  |  |  |  |  |
| 53 | Bureya |  |  | 1 |  |  |  |  |  |  |  |  |  |  |  | 1 |  |  |  |  |
| 54 | Levaya Bureya |  |  |  | 1 | 1 |  |  |  |  |  |  |  |  |  |  |  |  |  |  |
| 55 | Gobili |  |  |  | 1 |  |  |  |  |  |  | 1 |  |  |  |  |  |  |  |  |
| 56 | Ertukuli |  |  |  |  |  |  |  |  |  |  |  |  |  |  | 1 |  |  |  |  |
| 57 | Anui |  |  |  | 1 | 1 |  |  |  |  |  | 2 |  |  |  |  |  |  |  |  |
| 59 | Manoma | 1 |  |  |  |  |  |  |  |  |  | 1 | 1 |  |  | 1 |  |  |  |  |
| 61 | Khor |  | 1 |  |  | 2 |  |  |  |  |  | 2 |  |  |  | 1 |  |  |  |  |
| 62 | Suluk |  |  |  |  | 2 |  |  |  |  |  |  |  |  |  |  |  |  |  |  |
| 62 | Merek |  | 1 |  |  | 1 |  |  |  |  |  |  |  |  |  |  |  |  |  |  |
| 64 | Duki |  |  |  |  |  |  |  |  |  |  |  |  |  |  | 1 |  |  |  |  |
| 65 | Im |  | 1 |  |  | 1 |  |  |  |  |  |  |  |  |  |  |  |  |  |  |
| **Sakhalin** | | | | | | | | | | | | | | | | | | | | |
| 66 | Bol’shoi Vagis |  | 3 |  | 2 |  |  |  |  |  |  |  |  |  |  |  |  |  |  |  |
| **Shantar** | | | | | | | | | | | | | | | | | | | | |
| 69 | Yakshina |  |  |  |  | 1 |  |  |  |  |  |  |  |  |  |  |  |  |  |  |
| 70 | Bol’shoi Anaur |  |  |  |  | 4 |  |  |  |  |  |  |  |  |  |  |  |  |  |  |
|  |  | **Control Region Haplotype Number**  **Blunt-snouted lenok Sharp-snouted lenok** | | | | | | | | | | | | | | | | | | |
| **No.** | **Site/Basin** | CR1 | CR2 | CR3 | CR4 | CR5 | CR6 | CR7 | CR8 | CR9 | CR10 | CR11 | CR12 | CR13 | CR14 | CR15 | CR16 | CR17 | CR18 | CR19 |
| **Tugur** | | | | | | | | | | | | | | | | | | | | |
| 71 | Konin |  |  |  |  | 2 |  |  |  |  |  |  |  |  |  | 3 |  |  |  |  |
| **Uda** | | | | | | | | | | | | | | | | | | | | |
| 73 | Uda |  |  |  |  |  |  |  | 2 | 1 |  |  |  |  |  |  |  | 2 |  |  |
| 74 | Popkovskie lakes |  |  |  |  |  |  |  | 2 |  |  |  |  |  |  |  |  |  |  |  |
| **Primor’e** | | | | | | | | | | | | | | | | | | | | |
| 76 | Edinka |  |  |  |  |  |  |  |  |  | 1 |  |  |  |  |  |  |  |  |  |
| 78 | Beya |  |  |  |  |  |  |  |  |  | 1 |  |  |  |  |  |  |  |  |  |
|  | **TOTALS** | **1** | **6** | **18** | **5** | **15** | **3** | **2** | **6** | **1** | **2** | **6** | **7** | **10** | **23** | **25** | **9** | **2** | **2** | **6** |
