# Supplementary material for: The evolutionary history of sharp- and blunt-snouted lenok (Brachymystax lenok (Pallas, 1773)) and its implications for the paleo-hydrological history of Siberia
Source: BMC Evol Biol. 2008 Feb 6;8:40. doi: 10.1186/1471-2148-8-40 (PMC2275220; doi:10.1186/1471-2148-8-40)
Supplement: Additional File 4 — List of haplotypes and their frequencies for the ND1 gene analyzed across the populations sampled (N = 139 B. lenok individuals). [file 1471-2148-8-40-S4.DOC]

### Additional File 4 – List of haplotypes and their frequencies for the NADH-1 (N) gene analyzed for both blunt- and sharp-snouted lenok (*N* = 139). Haplotype numbers correspond to those in Figure 6. Site numbers correspond to those in Figure 1.

| **NADH-1 (ND) Haplotype Number**  **Blunt-snouted lenok Sharp-snouted lenok** | | | | | | | | | | | | | | | | | | | | | | | | | | | | | | | | | | | | |
| --- | --- | --- | --- | --- | --- | --- | --- | --- | --- | --- | --- | --- | --- | --- | --- | --- | --- | --- | --- | --- | --- | --- | --- | --- | --- | --- | --- | --- | --- | --- | --- | --- | --- | --- | --- | --- |
| **No.** | **Site/Basin** | 1 | 2 | 3 | 4 | 5 | 6 | 7 | 8 | 9 | 10 | 11 | 12 | 13 | 14 | 15 | 16 | 17 | 18 | 19 | 20 | 21 | 22 | 23 | 24 | 25 | 26 | 27 | 28 | 29 | 30 | 31 | 32 | 33 | 34 | 35 |
| **Ob** | | | | | | | | | | | | | | | | | | | | | | | | | | | | | | | | | | | | |
| 1 | Markakol’ |  |  |  |  |  |  |  |  |  |  |  |  |  |  |  |  |  |  |  |  |  |  |  |  |  |  |  |  |  |  |  |  |  | 4 |  |
| 2 | Kal’dzhir |  |  |  |  |  |  |  |  |  |  |  |  |  |  |  |  |  |  |  |  |  |  |  |  |  |  |  |  |  |  |  |  |  | 1 |  |
| 3 | Kara-Kaba |  |  |  |  |  |  |  |  |  |  |  |  |  |  |  |  |  |  |  |  |  |  |  |  |  |  |  |  |  |  |  |  |  | 2 |  |
| 4 | Pyzha |  |  |  | 1 | 1 |  |  |  |  |  |  |  |  |  |  |  |  |  |  |  |  |  |  |  |  |  |  |  |  |  |  |  |  |  |  |
| 5 | Biya |  |  |  |  | 3 |  |  |  |  |  |  |  |  |  |  |  |  |  |  |  |  |  |  |  |  |  |  |  |  |  |  |  |  |  |  |
| 6 | Mrassu |  |  |  |  |  |  |  |  |  |  |  |  |  |  |  |  |  | 1 |  |  |  |  |  |  |  |  |  |  |  |  |  |  |  |  |  |
| 7 | Kabyrza |  |  |  |  |  |  |  |  |  |  |  |  |  |  |  |  | 1 |  |  |  |  |  |  |  |  |  |  |  |  |  |  |  |  |  |  |
| 8 | Bol’shoi Kemchug |  |  |  |  |  |  |  |  |  |  |  | 1 |  |  |  |  | 1 |  |  |  |  |  |  |  |  |  |  |  |  |  |  |  |  |  |  |
| **Enisei (Selenga)** | | | | | | | | | | | | | | | | | | | | | | | | | | | | | | | | | | | | |
| 9 | Orkhon |  |  |  |  |  |  |  |  |  |  |  |  |  |  |  |  |  |  |  |  |  |  |  |  |  |  |  |  |  |  | 1 | 1 |  |  |  |
| 10 | Ero |  |  |  |  |  |  |  |  |  |  |  |  |  |  |  |  |  |  |  |  |  |  |  |  |  |  |  |  |  |  | 1 | 1 |  |  |  |
| 13 | Chovsgol |  |  |  |  |  |  |  |  |  |  |  |  |  |  |  |  |  |  |  |  |  |  |  |  |  |  |  |  |  |  |  | 1 |  |  |  |
| 14 | Khankhgol |  |  |  |  |  |  |  |  |  |  |  |  |  |  |  |  |  |  |  |  |  |  |  |  |  |  |  |  |  |  |  | 2 |  |  |  |
| **Ensisei (Baikal)** | | | | | | | | | | | | | | | | | | | | | | | | | | | | | | | | | | | | |
| 15 | Frolikha |  |  |  |  |  |  |  |  |  |  |  |  |  |  |  |  |  |  |  |  |  |  |  |  |  |  |  |  |  |  |  | 1 | 1 |  |  |
| **Enisei** | | | | | | | | | | | | | | | | | | | | | | | | | | | | | | | | | | | | |
| 18 | Kyzyl-Khem |  |  |  |  |  |  |  |  |  |  |  |  |  |  |  |  |  |  |  | 1 |  |  |  |  |  |  |  |  |  |  |  |  |  |  |  |
| 22 | Varlamovka |  |  |  |  |  |  |  |  |  |  |  |  |  |  |  |  |  |  |  | 1 | 4 |  |  |  |  |  |  |  |  |  |  |  |  |  |  |
| **Lena (upper)** | | | | | | | | | | | | | | | | | | | | | | | | | | | | | | | | | | | | |
| 23 | Nomama | 1 |  |  |  |  |  |  |  |  |  |  |  |  |  |  |  |  |  | 1 |  |  |  |  |  |  |  |  |  |  |  |  |  |  |  |  |
| 24 | Amudisa |  |  |  |  |  |  |  |  |  |  |  |  |  |  |  |  |  |  | 1 |  |  |  |  |  |  |  |  |  |  |  |  |  |  |  |  |
| 25 | Kalakan |  |  | 1 |  |  |  |  |  |  |  |  |  |  |  |  |  |  |  |  |  |  |  |  |  |  |  |  |  |  |  |  |  |  |  |  |
| 26 | Leprindokan |  |  |  |  |  |  |  |  |  |  |  |  |  |  |  |  |  |  | 2 |  |  |  |  |  |  |  |  |  |  |  |  |  |  |  |  |
| 27 | Kuanda | 4 |  |  |  |  |  |  |  |  |  |  |  |  |  |  |  |  |  | 1 |  |  |  | 1 |  |  |  |  |  |  |  |  |  |  |  |  |
| 31 | Amalyk | 2 |  |  |  |  |  |  |  |  |  |  |  |  |  |  |  |  |  |  |  |  |  |  |  |  |  |  |  |  |  |  |  |  |  |  |
| **Lena (middle)** | | | | | | | | | | | | | | | | | | | | | | | | | | | | | | | | | | | | |
| 32 | Bol’shoe Leprindo |  |  |  |  |  |  |  |  |  |  |  |  |  |  |  |  |  |  | 4 |  |  |  |  |  |  |  |  |  |  |  |  |  |  |  |  |
| 33 | Nameless lake | 2 |  |  |  |  |  |  |  |  |  |  |  |  |  |  |  |  |  |  |  |  |  |  |  |  |  |  |  |  |  |  |  |  |  |  |
| 35 | Utuk |  |  |  |  |  |  |  |  |  |  |  |  |  |  |  |  |  |  | 2 |  |  |  |  |  |  |  |  |  |  |  |  |  |  |  |  |
| 36 | Bol’shoe Toko |  |  |  |  |  |  |  |  |  |  |  |  |  |  |  |  |  |  | 2 |  |  |  |  |  | 1 |  |  |  |  |  |  |  |  |  |  |
| 37 | Yudoma |  |  |  |  |  |  |  |  |  |  |  |  |  |  |  |  |  |  |  | 2 |  |  |  |  |  |  |  |  |  |  |  |  |  |  |  |
| 38 | Kele | 4 |  |  |  |  |  |  |  |  |  |  |  |  |  |  |  |  |  | 1 | 2 |  |  |  |  |  |  |  |  |  |  |  |  |  |  | 1 |
| 39 | Vilui |  |  |  |  |  |  |  |  |  |  |  |  |  |  |  |  |  |  | 2 |  |  |  |  |  |  |  |  |  |  |  |  |  |  |  |  |
| 40 | Morkoka |  |  |  |  |  |  |  |  |  |  |  |  |  |  |  |  |  |  | 2 |  |  |  |  |  |  |  |  |  |  |  |  |  |  |  |  |
| 41 | Tuyng |  |  |  |  |  |  |  |  |  |  |  |  |  |  |  |  |  |  | 2 |  |  |  |  |  |  |  |  |  |  |  |  |  |  |  |  |
| **Lena (lower)** | | | | | | | | | | | | | | | | | | | | | | | | | | | | | | | | | | | | |
| 42 | Dyanyshka | 1 | 1 |  |  |  |  |  |  |  |  |  |  |  |  |  |  |  |  | 1 |  |  |  |  |  |  |  |  |  |  |  |  |  |  |  |  |
| 44 | Undyulyung |  |  |  |  |  |  |  |  |  |  |  |  |  |  |  |  |  |  | 3 |  |  |  |  |  |  |  |  |  |  |  |  |  |  |  |  |
| 45 | Tirekhtyakh |  |  |  |  |  |  |  |  |  |  |  |  |  |  |  |  |  |  | 2 |  |  |  |  |  |  |  |  |  |  |  |  |  |  |  |  |
| 46 | Sobolokh-Mayan |  |  |  |  |  |  |  |  |  |  |  |  |  |  |  |  |  |  |  |  |  |  | 2 |  |  |  |  |  |  |  |  |  |  |  |  |
| **Indigirka** | | | | | | | | | | | | | | | | | | | | | | | | | | | | | | | | | | | | |
| 48 | Indigirka |  |  |  |  |  |  |  |  |  |  |  |  |  |  |  |  |  |  | 3 |  |  |  |  |  |  |  |  |  |  |  |  |  |  |  |  |
| **Kolyma** | | | | | | | | | | | | | | | | | | | | | | | | | | | | | | | | | | | | |
| 50 | Popovka |  |  |  |  |  |  |  |  |  |  |  |  |  |  |  |  |  |  | 2 |  |  |  |  |  | 1 |  |  |  |  |  |  |  |  |  |  |
| **Amur** | | | | | | | | | | | | | | | | | | | | | | | | | | | | | | | | | | | | |
| 51 | Onon |  |  |  |  |  |  |  |  |  |  |  |  |  |  |  |  |  |  | 2 |  |  |  |  |  |  |  |  |  |  |  |  |  |  |  |  |
| 52 | Tok |  |  |  |  |  |  | 2 |  |  |  |  |  |  |  |  |  |  |  |  |  |  |  |  |  |  |  |  |  |  |  |  |  |  |  |  |
| 53 | Bureya |  |  |  |  |  |  |  |  |  | 1 |  |  |  |  |  |  |  |  | 1 |  |  |  |  |  |  |  |  |  |  |  |  |  |  |  |  |
| 55 | Gobili |  |  |  |  |  | 1 |  |  |  |  |  |  |  |  |  |  |  |  |  |  |  |  |  |  |  | 1 |  |  |  |  |  |  |  |  |  |
| 56 | Ertukuli |  |  |  |  |  |  |  |  |  |  |  |  |  |  |  |  |  |  |  |  |  | 1 |  |  |  |  |  |  |  |  |  |  |  |  |  |
| **NADH-1 (ND) Haplotype Number**  **Blunt-snouted lenok Sharp-snouted lenok** | | | | | | | | | | | | | | | | | | | | | | | | | | | | | | | | | | | | |
| **No.** | **Site/Basin** | 1 | 2 | 3 | 4 | 5 | 6 | 7 | 8 | 9 | 10 | 11 | 12 | 13 | 14 | 15 | 16 | 17 | 18 | 19 | 20 | 21 | 22 | 23 | 24 | 25 | 26 | 27 | 28 | 29 | 30 | 31 | 32 | 33 | 34 | 35 |
| 57 | Anui |  |  |  |  |  | 1 |  |  |  |  |  |  |  | 1 |  |  |  |  |  |  |  |  |  |  |  | 1 | 1 |  |  |  |  |  |  |  |  |
| 59 | Manoma |  |  |  |  |  | 1 |  |  |  |  |  |  |  |  |  |  |  |  |  |  |  | 1 |  |  |  | 2 |  |  |  |  |  |  |  |  |  |
| 61 | Khor |  |  |  |  |  | 3 |  |  | 2 |  |  |  |  |  |  |  |  |  | 1 |  |  | 1 |  |  |  |  |  |  |  |  |  |  |  |  |  |
| 62 | Suluk |  |  |  |  |  |  |  |  |  | 2 |  |  |  |  |  |  |  |  |  |  |  |  |  |  |  |  |  |  |  |  |  |  |  |  |  |
| 63 | Merek |  |  |  |  |  | 1 |  | 1 |  |  |  |  |  |  |  |  |  |  |  |  |  |  |  |  |  |  |  |  |  |  |  |  |  |  |  |
| 64 | Duki |  |  |  |  |  |  |  |  |  |  |  |  |  |  |  |  |  |  |  |  |  |  |  | 1 |  |  |  |  |  |  |  |  |  |  |  |
| 65 | Im |  |  |  |  |  | 2 |  |  |  |  |  |  |  |  |  |  |  |  |  |  |  |  |  |  |  |  |  |  |  |  |  |  |  |  |  |
| **Sakhalin** | | | | | | | | | | | | | | | | | | | | | | | | | | | | | | | | | | | | |
| 66 | Bol’shoi Vagis |  |  |  |  |  | 3 |  |  | 2 |  |  |  |  |  |  |  |  |  |  |  |  |  |  |  |  |  |  |  |  |  |  |  |  |  |  |
| **Shantar** | | | | | | | | | | | | | | | | | | | | | | | | | | | | | | | | | | | | |
| 69 | Yakshina |  |  |  |  |  | 1 |  |  |  |  |  |  |  |  |  |  |  |  |  |  |  |  |  |  |  |  |  |  |  |  |  |  |  |  |  |
| 70 | Bol’shoi Anaur |  |  |  |  |  | 3 |  |  |  |  | 1 |  |  |  |  |  |  |  |  |  |  |  |  |  |  |  |  |  |  |  |  |  |  |  |  |
| **Tugur** | | | | | | | | | | | | | | | | | | | | | | | | | | | | | | | | | | | | |
| 71 | Konin |  |  |  |  |  | 2 |  |  |  |  |  |  |  |  |  |  |  |  |  |  |  |  |  |  |  |  |  | 2 | 1 |  |  |  |  |  |  |
| **Uda** | | | | | | | | | | | | | | | | | | | | | | | | | | | | | | | | | | | | |
| 72 | Uda |  |  |  |  |  |  |  |  |  |  |  |  | 2 |  |  |  |  |  |  |  |  |  |  |  |  |  |  |  |  | 1 |  |  |  |  |  |
| 74 | Popkovskie lakes |  |  |  |  |  |  |  |  |  |  |  |  | 2 |  |  |  |  |  |  |  |  |  |  |  |  |  |  |  |  |  |  |  |  |  |  |
| **Primor’e** | | | | | | | | | | | | | | | | | | | | | | | | | | | | | | | | | | | | |
| 76 | Edinka |  |  |  |  |  |  |  |  |  |  |  |  |  |  |  | 1 |  |  |  |  |  |  |  |  |  |  |  |  |  |  |  |  |  |  |  |
| 78 | Beya |  |  |  |  |  |  |  |  |  |  |  |  |  |  | 1 |  |  |  |  |  |  |  |  |  |  |  |  |  |  |  |  |  |  |  |  |
|  | **TOTAL** | 13 | 1 | 1 | 1 | 4 | 18 | 2 | 1 | 4 | 3 | 1 | 1 | 4 | 1 | 1 | 1 | 2 | 1 | 35 | 6 | 4 | 3 | 3 | 1 | 2 | 4 | 1 | 2 | 1 | 1 | 2 | 6 | 1 | 7 | 1 |
