# Supplementary material for: The evolutionary history of sharp- and blunt-snouted lenok (Brachymystax lenok (Pallas, 1773)) and its implications for the paleo-hydrological history of Siberia
Source: BMC Evol Biol. 2008 Feb 6;8:40. doi: 10.1186/1471-2148-8-40 (PMC2275220; doi:10.1186/1471-2148-8-40)
Supplement: Additional File 10 — Details for amplification, sequencing, primers and morphological measurements used in this study. [file 1471-2148-8-40-S10.DOC]

**Additional File 9 - Details for amplification, sequencing, primers and morphological measurements used in this study**

*Amplification and sequencing*

For samples that revealed degraded DNA, one new primer (Bl578R- CGACCTTGTAGACTTCTTTGC) was used in combination with LRBT-25 to amplify the left-domain of the CR only. Again, new primers were designed to amplify shorter fragments (300-600 bp) of the ND1: BlNDF1– GCACAAACTATTTCCTACGA; BlNDF2- TTTCGCGATTTAATGTAGAA; BlNDF3- TAAAGAACCAGTTCGACCAT; BlNDR1- CGTTGAAGGTTTGAAGTGTG; BlNDR2- GGCAGCTTTTGTTATGAGA BlNDR3- TGGTCGAACTGGTTCTTTAA. The combinations were as follows: BlFNDF+BlNDR1; BlNDF1+BlNDR; BlNDF1+BlNDR2; BlNDF2+BlNDR; BlNDF+BlNDR3; BlNDF3+BlNDR1.

*Microsatellites*

Primer sequences and repeat motifs for the di-nucleotide loci are: BleDi3F-CGCAGACCATCTTTTGATTGT, BleDi3R-GTAGGGTTGTGTTGGCCATGT, (CT)_7_; BleDi4F-GCTCCCCCTGTGCCATCTGGT, BleDi4R-CCACTGCCATCAGCACTCTTG, (GT)_21_; BleDi5F-AAGGGAGTGAATGATAGTTGG, BleDi5R-TTTGTTCAGTATAGATGGGTG, (AT)_4_(AC)_21_(AT)_3_(AC)_4_; BleDi9F-TACAAACGATGTCAAAAAAGG, BleDi9R-GGTGCCATTCACCCCCTCCAG, (GT)_11_. Primer annealing was: 55°C for BleDi3, 57°C for Bledi4, 5, and 59°C for the BleDi9; 15 s.

*Morphological Measurements*

**Measurements included**: fork length (FL); head length (c); head depth at the level of occiput (Hc); head depth at the level of the center of the eye (hc); snout length (ao); interorbital width (io); upper jaw length (lrm); length of the maxilla (lmx) ; upper jaw width (hmx); lower jaw length (lmd); maximum body depth (H); least depth of the caudal peduncle (h); predorsal length (aD); preventral length (aV); preanal length (aA); pectoral origin to pelvic base length (P-V), length of caudal peduncle (pl).

**Meristic characters included**: number of gill rakers on the left side (sb1), number of branchiostegal rays on the left side (rb1) and right side (rb2), number of branched rays in the dorsal fin (D); in the anal fin (A); and number of pyloric caeca (pc). We counted all gill rakers (including the smallest terminal ones); the last bifurcated ray in the dorsal and anal fins was counted as one, if two branches had the same base. In the samples represented by fixed heads (from localities 11-13, 17, 22, 23, 42, 46, 50, 67, 68, 76) only c, Hc, hc, ao, io, lrm, lmx, hmx, lmd, sb1, rb1, and rb2 were measured.

**Osteological characters included**: skull base length (Lcr); skull depth at the level of the lateral ethmoids (Het), at the level of the sphenotics (Hsp), at the level of the supraoccipitals (Hsup); skull width at the level of the lateral ethmoids (Set), at the level of the sphenotics (Ssp), at the level of the pterotics (Spt), width and length of the opercle (Op.W and Op.L), of the subopercle (Sop.W and Sop.L), of the preopercle (Pop.W and Pop.L), of the supraethmoid (Seth.W and Seth.L), of the vomer (V.W and V.L), width, depth, and length of the glossohyal (G.W, G.H, and G.L), width of the anterior part of the parasphenoid (P.W1), width of the parasphenoid at the level of the lateral processes (P.W2), minimum width of the parasphenoid (P.Wm), length of the parasphenoid (P.L), depth and length of the premaxilla (Pm.H and Pm.L), of the dentary (D.H and D.L), width and length of the maxilla (Mx.W and Mx.L), of the frontal (F.W and F.L), of the hyomandibular (H.W and H.L). We also counted number of teeth on the vomer (V.d) and number of teeth on the left side of the glossohyal (G.d1). In samples from the Duki River only the skull, the opercle, the preopercle, the supraethmoid, and the maxilla were measured.
