# Supplementary material for: The evolutionary history of sharp- and blunt-snouted lenok (Brachymystax lenok (Pallas, 1773)) and its implications for the paleo-hydrological history of Siberia
Source: BMC Evol Biol. 2008 Feb 6;8:40. doi: 10.1186/1471-2148-8-40 (PMC2275220; doi:10.1186/1471-2148-8-40)
Supplement: Additional File 12 — Extended list of acknowledgements. [file 1471-2148-8-40-S12.DOC]

**Additional File 10 – Details for acknowledgements**

*Acknowledgements*

We thank colleagues for providing samples of *B. lenok* for genetic and/or morphological analysis: M. Baimukanov (Kazakh Institute of Fish Culture, Ministry for Science and Higher Educatiin of Kazakhstan, Almaty, Kazakhstan) – Markakol’ (genetics), Kara-Kaba; M.V. Mina (Institute of Developmental Biology, RAS, Moscow) – Markakol’(partly morphology), Kal’dzhir (genetics, partly morphology); B.E. Bogdanov, I.B. Knizhin, A.N. Matveev, V.P. Samusenok (Irkutsk State University, Russia) – Biya, Frolikha, Onon; Yu.Yu. Dgebuadze (Institute of Ecology and Evolution, RAS, Moscow) and V.I. Lapin (Moscow State University) – Ero, Ider, Delger-Muren; P.Ya. Tugarina (Irkutsk State University) – Chovsgol, Khankhgol; N. Troitskaya (Barguzin State Preserve, Russia) – Bol’shaya; D. Kuksina (Tuva State University, Kyzyl, Russia) – Kyzyl-Khem; B.I. Sheftel’, A. Panaiotidi (Institute of Ecology and Evolution, RAS), F.N. Shkil’, S.G. Dmitriev (Institute of Developmental Biology, RAS) – Varlamovka; Wild Salmon Center (Portlend, USA), M.B. Skopets (Institute of Biological Problems of the North, FEB RAS, Magadan, Russia) – Yudoma, Konin, Im; M.Yu. Pichugin (Moscow State University) and M.B.Skopets – Yakshina (genetics), Bol. Anaur (genetics); A.F. Kirillov (Institute of Applied Ecology of the North, Republic Sakha (Yakutia) AS, Yakutsk, Russia) – Morkoka (partly morphology), Undyulyung (genetics, partly morphology), Kele, Tyung, Dyanyshka, Sobolokh-Mayan, Indigirka, Popovka; A.I. Pozdnyakov (Republic Sakha (Yakutia), Yakutsk, Russia) – Vilui; V.N. Zaitsev (Moscow State University) – Tok (partly morphology), Uda (partly morphology); A.L. Antonov (Institute of Aquatic and Ecological Problems, FEB RAS, Khabarovsk, Russia) – Bureya, Levaya Bureya, Suluk, Merek, P. perryi; V.A. Maksimov, M.A. Gruzdeva, D.V. Politov, N.Yu.Gordon (Moscow State University) – Anui (partly morphology), Uda (genetics, partly morphology), Popkovskie lakes; P.K. Gudkov and Yu.I. Dudnik (Pacific Institute of Fisheries and Oceanography, Sakhalin Section, Yuzhnosakhalinsk) – Bol’shoi Vagis, Ten’gy, Pyrki; I.Z. Parpura (Pacific Institute of Fisheries and Oceanography, Vladivostok) – Edinka, Samarga (partly morphology). We thank S.G. Afanas’ev, N.V. Alekseyeva, A.S. Alekseyev, A.L. Antonov, M. Baimukanov, I.I. Berkutov, A.I. Bokut’, V.V. Buldygerov, W. Davis, N.Ya. Demidov, S.Yu. Egorov, M.A. Gruzdeva, A.K. Karpov, Yu.I. Kharchenkov, A.Ya. Khramov, A.M. Kirdyapkin, E.F. Kirik, A. Klintsov, I.B. Knizhin, V.V. Korepanov, M.N. Krivosheev, O.N. Lisitsyna, V.A. Maksimov , S.V. Maslov, A.N. Matveev, Yu.I. Mederov, M.V. Mina, A. Moravetskii, S. Morzunov, T. Morzunova, I.Z. Parpura, D.A. Pavlov, M.Yu. Pichugin, A.I. Pozdnyakov, D. Proebstel, V.V. Pulyarov, V.P. Samusenok, K.A. Savvaitova, A. Yu.Shanin, F.N. Shkil’, A.A. Sokolov, Yu.I. Tsarev, A.I.Vokin, A.L. Yur’ev, V.N. Zaitsev, A.A. Zyuzin for field assistance.
